# Dynamic Network- and Microcellular Architecture-Driven Biomass Elastomer toward Sustainable and Versatile Soft Electronics

**DOI:** 10.1007/s40820-025-01942-7

**Published:** 2025-12-13

**Authors:** Shanqiu Liu, Yi Shen, Yizhen Li, Yunjie Mo, Enze Yu, Taotao Ge, Ping Li, Jingguo Li

**Affiliations:** 1 Institute for Frontiers and Interdisciplinary Science, Zhejiang University of Technology, Hangzhou, 310014 People’s Republic of China; 2 State Key Laboratory of Advanced Environmental Technology, Department of Environmental Science and Engineering, University of Science and Technology of China, Hefei, 230026 People’s Republic of China; 3 School of Materials Science and Engineering, Xi’an Jiaotong University, Xi’an, 710049 People’s Republic of China

**Keywords:** Bio-based conductive elastomers, Dynamic covalent chemistry, Micromechanical sensitivity, Soft electronics

## Abstract

Conductive elastomers combining micromechanical sensitivity, lightweight adaptability, and environmental sustainability are critically needed for advanced flexible electronics requiring precise responsiveness and long-term wearability; however, the integration of these properties remains a significant challenge. Here, we present a biomass-derived conductive elastomer featuring a rationally engineered dynamic crosslinked network integrated with a tunable microporous architecture. This structural design imparts pronounced micromechanical sensitivity, an ultralow density (~ 0.25 g cm^−3^), and superior mechanical compliance for adaptive deformation. Moreover, the unique micro-spring effect derived from the porous architecture ensures exceptional stretchability (> 500% elongation at break) and superior resilience, delivering immediate and stable electrical response under both subtle (< 1%) and large (> 200%) mechanical stimuli. Intrinsic dynamic interactions endow the elastomer with efficient room temperature self-healing and complete recyclability without compromising performance. First-principles simulations clarify the mechanisms behind micropore formation and the resulting functionality. Beyond its facile and mild fabrication process, this work establishes a scalable route toward high-performance, sustainable conductive elastomers tailored for next-generation soft electronics.

**Supplementary Information:**

The online version contains supplementary material available at 10.1007/s40820-025-01942-7.

## Highlights

- Biomass-derived conductive elastomer featuring dynamic networks and microporous architecture enables ultralight and highly mechanosensitive soft electronics.
- Micro-spring-like porous structure imparts excellent stretchability, superior resilience, and rapid and precise electrical responsiveness under subtle and large mechanical stimuli.
- Intrinsic dynamic interactions enable efficient room temperature self-healing and full recyclability, promoting sustainable and scalable fabrication of advanced flexible electronics.

**Supplementary Information:**

The online version contains supplementary material available at 10.1007/s40820-025-01942-7.

## Graphical abstract

**Figure:**
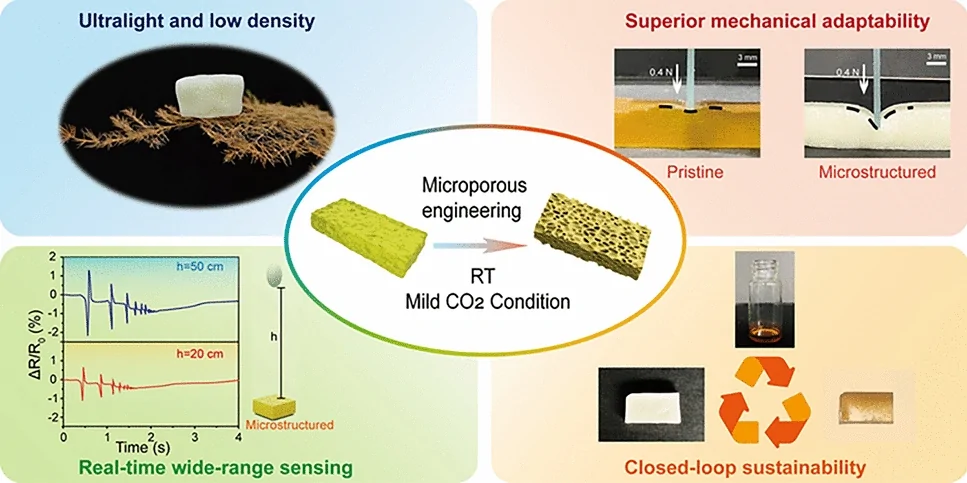

## Introduction

Conductive elastomers have emerged as essential building blocks in next-generation soft electronic devices, playing pivotal roles in wearable sensors, intelligent human–machine interfaces, advanced health monitoring, and soft robotics [1–4]. The unique integration of mechanical flexibility and electrical conductivity allows these materials to conform seamlessly to dynamic biological interfaces, thus enabling precise, continuous tracking of physiological and mechanical stimuli [5–8]. However, despite recent advancements, conventional conductive elastomers still face notable performance limitations, particularly inadequate sensitivity to subtle mechanical signals, such as gentle pressure or light contact forces, and sluggish dynamic responsiveness, significantly restricting their capacity to accurately capture transient or delicate changes [9–11]. Moreover, their intrinsically dense structures result in increased material weight and reduced mechanical compliance, diminishing user comfort and limiting their practical suitability for prolonged wearable applications [12]. Coupled with environmental concerns arising from reliance on petrochemical-derived polymers [13–15], these limitations highlight an urgent demand for a new generation of conductive elastomers offering high sensitivity, lightweight architecture, mechanical adaptability, and environmental sustainability [16, 17].

Biomass-derived conductive elastomers have recently gained considerable attention as sustainable and renewable alternatives to conventional synthetic elastomers, owing to their inherent degradability, biocompatibility, and abundant sourcing from renewable biomass components [18, 19]. Numerous efforts have explored plant oils, lignin, cellulose derivatives, and other biomass-derived small molecules to fabricate flexible, conductive elastomeric matrices with reduced environmental impact [20–24]. Enhancing the electromechanical sensitivity of these elastomers generally involves incorporating conductive fillers such as carbon-based nanomaterials, metallic nanostructures, and intrinsically conducting polymers [25–32]. For example, Zhang et al. reported a self-healing conductive elastomer based on polysiloxane grafted with lipoic acid, where dynamic S–Au interactions endowed the material with enhanced stretchability and near-infrared-assisted healing, along with strain-sensing capabilities for joint and muscle movement monitoring [33]. Hou et al. developed solvent-free conductive gels via supramolecular assembly of sodium methylallyl sulfonate-functionalized poly(lipoic acid) chains and polyaniline nanorods, achieving dual-mode strain and temperature sensing with skin-like compliance [34]. Woo et al. constructed a closed-loop recyclable composite by crosslinking biomass-derived poly(ethyl lipoate) with multi-walled carbon nanotubes [35]. This system demonstrated rapid and complete acid-triggered depolymerization and retained mechanical/electrical performance across multiple recycling cycles, highlighting its potential for durable and eco-friendly strain-sensing applications. Although these strategies have achieved significant improvements in conductivity, mechanical strength, and sensing performance, they commonly rely on rigid filler incorporation. Such approaches inherently cause interfacial instability during cyclic deformation, leading to signal drift, mechanical fatigue, and premature functional degradation [36–39]. More critically, previous designs often prioritize electrical and mechanical performance at the expense of structural compliance and density optimization, leading to elastomers with increased mass and rigidity, as well as diminished deformability, which significantly compromises their mechanical sensitivity, responsiveness to dynamic loading, and overall comfort in wearable applications [40, 41].

Lightweight structural strategies—particularly porous architecture engineering—are critical for enhancing mechanical compliance, electromechanical sensitivity, and user comfort in flexible electronics [42–44]. Surprisingly, this principle has rarely been explored in biomass-based conductive elastomers [12]. Traditional lightweighting methods, such as supercritical CO_2_ foaming, chemical etching, freeze-drying, and additive manufacturing, typically require harsh processing conditions. Elevated temperatures, high pressures, and aggressive solvents used in these methods often degrade thermally sensitive biomass-based matrices or disrupt their delicate molecular structures [45–52]. Such incompatibility inevitably compromises structural integrity, mechanical robustness, and electromechanical performance. Therefore, developing a mild, efficient, and sustainable strategy that enables the construction of tunable porous architectures while simultaneously achieving high sensing performance and mechanical adaptability remains an unresolved challenge in biomass-derived conductive elastomers.

In this work, we devised a rational molecular design strategy for fabricating biomass-based conductive elastomers featuring tunable microporous architectures under mild, room temperature CO_2_ conditions. Our approach strategically leverages dynamic polymer networks, polarity modulation, and intermolecular lubrication effects through synergistic integration of biomass-derived lipoic acid (LA)-based matrices, nano-reinforced multi-point interactions, and hydrogen bonding ionic species. This engineered framework significantly enhances molecular mobility, reduces the energy barrier for pore formation, yielding a lightweight elastomer with a uniform and tunable microporous structure. Importantly, the porous architecture provides pronounced localized strain amplification, resulting in markedly enhanced sensitivity to subtle mechanical stimuli and improved mechanical adaptability under both static and dynamic loads. Further, the spring-like behavior originates from the synergy between the microcellular architecture and the dynamic crosslinked network, enabling efficient energy storage and release for rapid elastic recovery and fast electromechanical response. Molecular-level insights from first-principles simulations further reveal the synergistic roles of dynamic interactions and microscale pore formation mechanisms underlying these enhanced performances. With intrinsic self-healing and excellent recyclability, this engineered elastomeric platform represents a significant advancement in sustainable soft electronic materials, offering compelling new opportunities for wearable sensors, intelligent human–machine interfaces, and next-generation soft robotics.

## Experimental Section

### Material

α-Lipoic acid (LA, AR) and oxalyl chloride (AR) were procured from Adamas®beta. Triethylamine (AR) and N,N-dimethylformamide (DMF, AR) were purchased from Aladdin. Aminopropyltriethoxysilane (APTES, AR) and ethyl sulfate-1-methyl-3-ethylimidazole (EMIES, AR) were sourced from Macklin. Dichloromethane (AR) and ethanol (AR) were obtained from Titan Co., Ltd. All SiO_2_ nanoparticles were acquired from Zhuotai New Materials Technology Co., Ltd. Unless otherwise specified, all regents were used as received.

### Synthesis of the SiO_2_-LA Nanoparticles

3.0 g of the SiO_2_ nanoparticles were ultrasonically dispersed in 50 mL of anhydrous ethanol. The mixture was heated in an oil bath at 60 °C, followed by the addition of 5 mL of APTES under reflux with vigorous stirring for 6 h. The resulting turbid suspension was centrifuged, and the supernatant was discarded to obtain a white powdery solid, SiO_2_-NH_2_. The product was washed three times with ethanol and dichloromethane and ultrasonically dispersed in dichloromethane for further use.

5.0 g of LA was dissolved in 20 mL of dichloromethane in a flask, followed by the addition of 3.1 mL of oxalyl chloride under rapid stirring at room temperature. After stirring for 3 min, 150 µL of DMF were added, and the reaction was allowed to proceed for 4 h. The resulting solution was evaporated to dryness, and the brown crystalline residue was dissolved in a small amount of dichloromethane. This solution was then slowly added dropwise to the SiO_2_-NH₂ dispersion in dichloromethane under ice-water bath conditions and allowed to react overnight. The resultant dark brown turbid suspension was centrifuged, washed three times with dichloromethane, and dried to obtain a brown powdery solid, SiO_2_-LA.

### Synthesis of the Elastic Composites

1.0 g of LA was mixed with a certain amount of SiO_2_-LA and subjected to rapid stirring in an oil bath at 150 °C for 10 min, yielding a yellowish-brown transparent liquid. Subsequently, 0.22 g (184 μL) of ionic liquid EMIES was added, and the mixture was stirred rapidly for 15 min. The resulting yellowish-brown transparent liquid was then poured into a mold and allowed to cool at room temperature to form the elastic composites.

### Microporous Structuring Procedure

The prepared composites were cut into specimens measuring 15.0 mm × 5.0 mm and placed in a high-pressure chamber, where they were saturated with CO_2_ at 2.0 MPa and room temperature for 6 h. Upon rapid depressurization, the composites underwent a structural transformation, forming a microporous architecture at ambient conditions. All samples were then left in air at room temperature for at least 6 h to complete the structural evolution prior to further characterization. For comparison, additional experiments were also performed at CO_2_ pressures of 1 and 5 MPa.

### Characterizations

Attenuated total reflectance Fourier-transform infrared (ATR-FTIR) spectra were recorded using a Nicolet 6700 spectrometer in the range of 4000–650 cm^−1^, and the Fourier-transform infrared (FTIR) spectra were recorded in the range of 4000–500 cm^−1^. Ultraviolet–visible (UV–Vis) absorption spectra of the composite and lipoic acid ethanol solution were measured using a LAMBDA 1050 + UV–Vis-NIR spectrophotometer (PerkinElmer, USA) in the wavelength range of 500–250 nm. X-ray photoelectron spectroscopy (XPS) measurements were performed using a Thermo Scientific K-Alpha spectrometer (Thermo Fisher Scientific, USA) with Al Kα radiation (12 kV).

The microporous morphology of the composite was examined using a VEGA3 scanning electron microscope (TESCAN, Czech Republic) operated at an accelerating voltage of 10 kV. Prior to imaging, samples were immersed in liquid nitrogen for 20 min to induce brittle fracture and expose internal structures. Nanoparticles were imaged and subjected to elemental analysis using an S-4700 field-emission scanning electron microscope (HITACHI, Japan) equipped with energy-dispersive X-ray spectroscopy (EDS), operated at an accelerating voltage of 15 kV.

### Calculation of Density and Porosity

The density (ρ) of the composite, both before and after microporous structuring, was calculated by measuring the mass (m) and volume (V) according to Eq. 1:

1 $$\rho =\frac{m}{V}$$

The porosity (P) was subsequently determined based on the density values before (ρ_1_) and after (ρ_2_) the structural transformation, using Eq. 2:

2 $$P=\frac{{\rho }_{1}-{\rho }_{2}}{{\rho }_{1}}$$

### Calculation of Pore Size and Pore Density

Pore size and density were determined from SEM images of the cross sections of the microporous samples. The pore density (Nv) was approximated using the Kumar method, [53] which does not require direct measurement of individual pore diameters. Instead, the number of pores (n) within a given SEM image area (A) was counted, and the image magnification (M) was recorded. The pore density Nv was then calculated using Eq. 3:

3 $$Nv = \left( {\frac{{nM^{2} }}{A}} \right)^{{\frac{3}{2}}}$$

### Mechanical Tests

Tensile tests of the microporous and dense composites were measured using a universal testing machine (Instron 5567, USA) at a crosshead speed of 50 mm min^−1^. Specimens were prepared with dimensions of 35.0 mm × 5.0 mm × 5.0 mm. Compression tests were performed on cylindrical samples (diameter: 20 mm, height: 20 mm) at a displacement rate of 20 mm min^−1^. The initial length (L_0_) and the length after unloading (L) were recorded during cyclic testing. The tensile recovery ratio (R_t_) is calculated according to Eq. 4:

4 $${R}_{t}=1-\frac{L-{L}_{0}}{{L}_{0}}$$

The compressive recovery ratio (R_c_) is determined by Eq. 5:

5 $${R}_{c}=1-\frac{{L}_{0}-L}{{L}_{0}}$$

Material toughness was determined by numerical integration of the tensile stress–strain curve up to fracture.

### Electrical Tests

The relative resistance changes of the composites under applied strain, pressure, and temperature were evaluated using a digital multimeter (Keithley 2400) and an electrochemical workstation (CHI-630E), with an output voltage of 3.6 V. Composites with dimensions of 35.0 mm × 10.0 mm × 5.0 mm were used to construct wearable sensors designed for monitoring joint movements. The ends of the composite were connected to copper wires and then directly affixed to the testing leads for motion detection via the electrochemical workstation. The relative resistance is calculated using Eq. 6:

6 $$\frac{\Delta R}{{R}_{0}}=\frac{R-{R}_{0}}{{R}_{0}}\times 100\%$$

where R is the resistance of the elongated composite, and R₀ is the original resistance of the composite. The gauge factor (GF) was employed to quantify the strain sensitivity of both the dense and microporous composites, calculated using Eq. 7:

7 $$GF=\frac{\Delta R}{{R}_{0}}\times \frac{1}{\epsilon }$$

ε represents the applied strain.

### Self-Healing Tests

Samples with dimensions of 35.0 mm × 5.0 mm × 5.0 mm were cut into two equal parts, which were then brought into contact without external stimuli to undergo a series of healing times. The healed samples were evaluated using a tensile testing apparatus, and the self-healing efficiency was determined as the ratio of the tensile strain at the break point between the original and healed samples.

### Calculation Method

Based on the framework of the density-functional theory (DFT), the first-principles calculations were performed using the Vienna Ab initio Simulation Package (VASP) [54–56], with the Perdew–Burke–Ernzerhof (PBE) functionals in the framework of generalized gradient approximation (GGA) [57]. The energy cut-off for the plane-wave expansion was established as 500 eV [58]. The force and total energy convergence criterion are set to be -0.01 eV Å^−1^ and 10^−6^ eV, respectively [59, 60]. The binding energy (adsorption energy) is defined as Eqs. 8 and 9:

8 $${E}_{b}={E}_{tot}-{E}_{A}-{E}_{B}$$

9 $${E}_{ad}={E}_{tot}-{E}_{A}-{E}_{{co}_{2}}$$

E_tot_, E_A_, E_B_, and E_CO2_ are the total energies of system, the A, the B (A and B represent COOH, CONH, and EMIES), and CO_2_, respectively.

### Recycling of the Composites

Recycling was achieved by heating the used microporous composite at 150 °C under stirring at 600 rpm for 10 min, resulting in its transition into a yellowish-brown transparent liquid. This regenerated liquid was then cast into a mold and cooled to room temperature to form a new elastomer. Subsequently, the mild CO_2_-induced structuring process, as described earlier, was reapplied to reintroduce the microporous architecture in the regenerated material.

### Recycling of the SiO_2_-LA

The recycling process involves dissolving the used microporous composite in excess ethanol, followed by centrifugation at 10,000 rpm for 30 min. The recovered SiO_2_-LA nanoparticles were washed three times with anhydrous ethanol and vacuum-dried. These recycled nanoparticles were subsequently recombined with an ethanol solution of lipoic acid to prepare a new composite, which could then undergo the previously described CO_2_-induced microporous structuring process.

## Results and Discussion

### Materials Design and Characterization

**Fig. 1:**
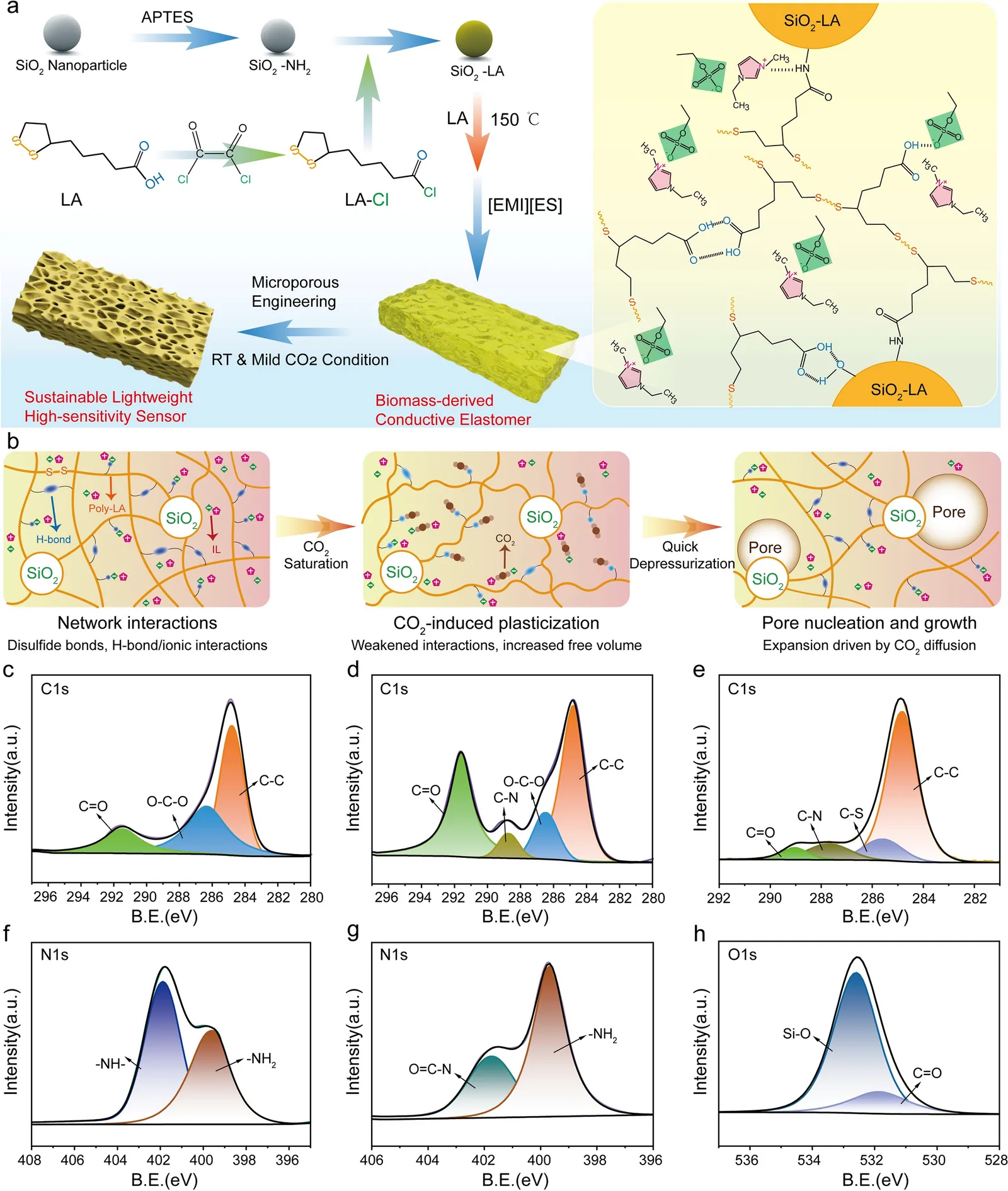
Synthesis and characterization. **a** Schematic illustration of the fabrication process for the functional microporous composite featuring lightweight architecture and high sensing sensitivity. **b** Schematic illustration of CO_2_-induced plasticization and the associated pore nucleation and growth in the composite. **c** C 1*s* XPS spectrum of pristine SiO_2_ nanoparticles. **d** C 1*s* XPS spectrum of amino-functionalized SiO_2_ (SiO_2_-NH_2_). **e** C 1*s* XPS spectrum of LA-modified SiO_2_ (SiO_2_-LA). **f** N 1*s* XPS spectrum of SiO_2_-NH_2_. **g** N 1*s* XPS spectrum of SiO_2_-LA. **h** O 1*s* XPS spectrum of SiO_2_-LA

The preparation process for the biomass-derived conductive elastomer is schematically illustrated in Fig. 1a, b. To synthesize LA-functionalized silica nanoparticles (SiO_2_-LA), a mild reaction between amino groups and acyl chlorides was employed. First, 3-aminopropyltriethoxysilane (APTES) was reacted with hydroxyl groups on the nanoparticle surface for amino-functionalization. Lipoic acid, pre-activated using oxalyl chloride, was then grafted onto the amino-functionalized nanoparticles. Scanning electron microscopy (SEM) (Fig. S1) indicated no significant changes in surface morphology or size after modification. Energy dispersive spectroscopy (EDS) analysis (Fig. S2a) revealed a notable increase in nitrogen content following APTES modification, confirming successful amino-functionalization. The appearance of sulfur in the EDS spectrum after LA grafting confirmed the introduction of 1,2-dithiolane rings on the nanoparticle surface. Fourier-transform infrared (FTIR) spectroscopy of SiO_2_-LA showed characteristic peaks at 1250 and 1750 cm⁻^1^, corresponding to amide groups, further validating the chemical modification (Fig. S2b) [45, 61]. XPS analysis provided additional evidence for these molecular transformations (Fig. 1c-h). After ATPES amination, the SiO_2_ nanoparticles exhibit a clear C–N bonding peak in the C 1*s* spectrum at approximately 288.7 eV, accompanied by a characteristic primary amine (–NH_2_) peak at 399.5 eV and an imine (–C=N–) peak at 401.9 eV in the N 1*s* region, collectively indicating successful surface amination of the nanoparticles [62]. Upon subsequent modification with lipoic acid, the presence of a distinct C–S bond at 285.6 eV (C 1*s*) and an amide nitrogen peak (–N–C=O) at 401.8 eV (N 1*s*) are clearly observed [63]. These nitrogen signals, corroborated by the corresponding carbon spectra, further confirm the successful amidation reaction between the amino-functionalized nanoparticles and lipoic acid.

Next, LA and SiO_2_-LA nanoparticles were mixed and heated to 150 °C, forming a transparent yellow–brown liquid. Upon vigorous stirring with ionic liquid (IL, 1-ethyl-3-methylimidazolium ethylsulfate), a solid elastic composite with a crosslinked structure formed at room temperature via dynamic covalent bond self-assembly (Figs. 1a and S3). This process utilized the ring-opening polymerization of disulfide bonds in LA and SiO_2_-LA under thermal conditions, forming the poly-LA network. Notably, the SiO_2_-LA nanoparticles served as nanoscale multi-point crosslinking sites, facilitating dynamic crosslinking with the poly-LA network through disulfide bonds. This multi-point interaction not only enhances the structural integrity by forming reversible disulfide bonds with the poly-LA chains, but also mitigate interfacial defects frequently encountered in conventional rigid filler-matrix composites. The incorporation of IL formed hydrogen bonds and electrostatic interactions with carboxyl groups on the poly-LA network, further stabilizing the network and providing conductive ion pathways [64, 65]. Additionally, SEM imaging and EDS mapping of the composite cross sections (Fig. S4) confirm that SiO_2_-LA nanoparticles are uniformly dispersed within the polymer matrix without visible agglomeration. The homogeneous Si distribution in the EDS maps further corroborates their uniform distribution. UV–Vis spectroscopy (Fig. S5) showed the disappearance of the characteristic absorption peak at ~ 330 nm (present in pure LA), indicating the cleavage and reformation of disulfide bonds, essential for the dynamic crosslinked poly-LA network [66, 67].

**Fig. 2:**
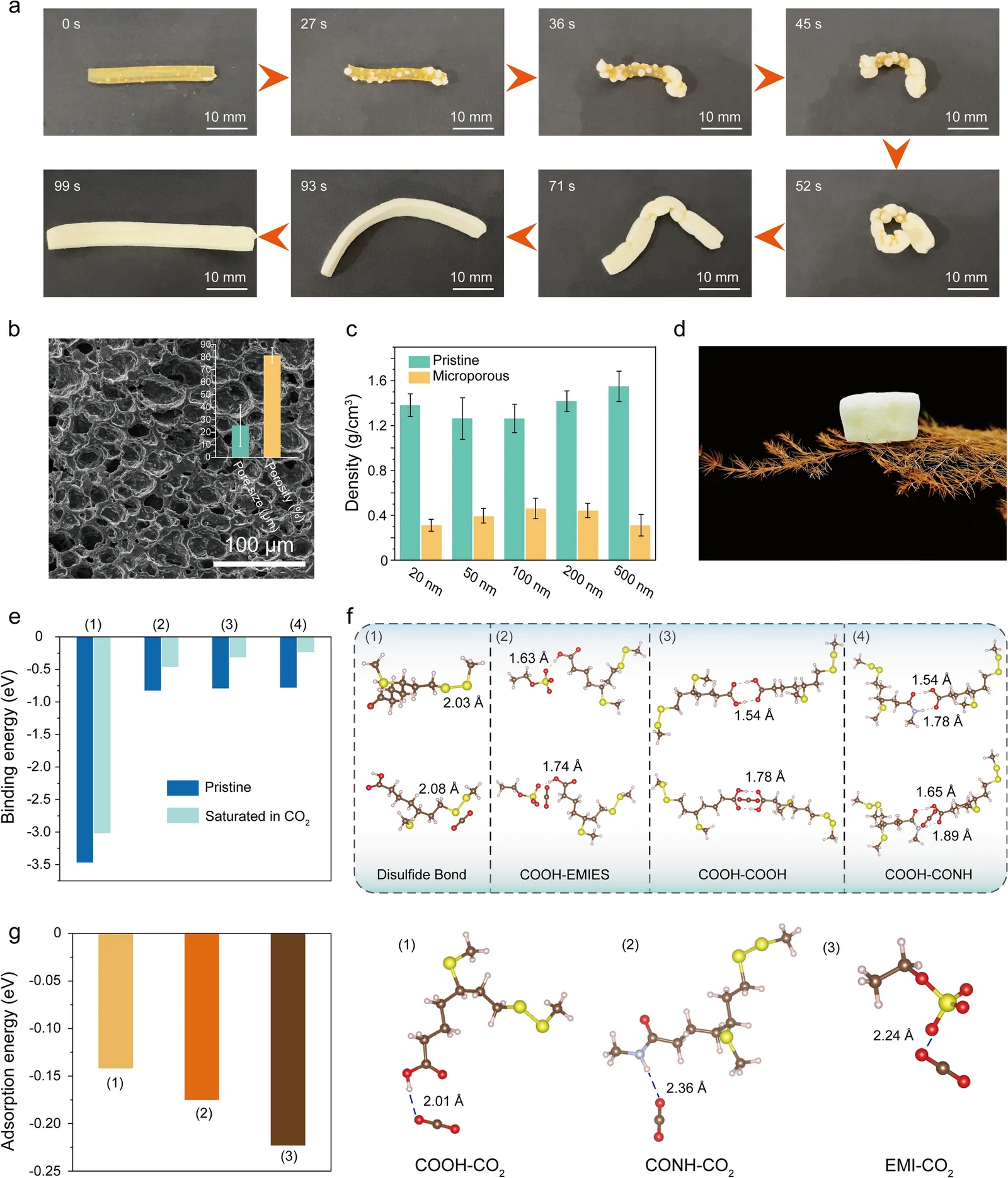
Microporosity engineering and mechanistic insights.** a** Visual evolution of CO_2_-induced microporosity under ambient conditions: from dense to microporous architecture. **b** SEM image illustrating the uniform microporous morphology of the composite formulated with 1 wt% 20 nm nanoparticles and 20 wt% IL; the inset highlights representative pore size and porosity. **c** Density comparison before and after microporous structuring for composites containing 1 wt% nanoparticles and 20 wt% IL. **d** Demonstration of ultralight characteristics via minimal mechanical support. **e** Calculated interaction energies before and after CO_2_ infusion. **f** Schematic of molecular interactions and bond lengths, with top and bottom panels depicting the composite before and after CO_2_ infusion, respectively. **g** Binding energies of CO_2_ with composite components

To induce microporosity under mild conditions, the composite was exposed to CO_2_ at room temperature and relatively low pressure (2.0 MPa). During CO_2_ impregnation, pressurized CO_2_ dissolves and diffuses into the polymer matrix, inducing plasticization by weakening hydrogen bonding and ionic interactions and increasing the intermolecular free volume, which enhances chain mobility (Fig. 1b). Meanwhile, nanoparticles serve as preferential sites for heterogeneous nucleation, thereby lowering the energy barrier for pore formation. Upon depressurization, dissolved CO_2_ gradually diffused out, providing the driving force for pore nucleation and growth. Accompanied by this controlled release, the composite visibly expanded at ambient conditions, transitioning slowly from a transparent yellow state to an opaque ivory appearance (Figs. 2a and S6). This transformation was facilitated by the dynamic molecular rearrangements and local plasticization effects from CO_2_, allowing micropore formation uniformly throughout the matrix without requiring elevated temperatures or harsh conditions. SEM analyses (Figs. 2b and S7) revealed well-defined microporous structures markedly distinct from the initial dense morphology. Following microporous structuring, the composite exhibited a significant volume expansion of approximately 3–5 times, corresponding to a porosity of 70%-80% and a notable reduction in density to merely 20%-30% of its initial dense state (Figs. 2c and S8). The resulting composite, visually highlighted in Fig. 2d by its effortless support on delicate substrates, demonstrates substantial promise for lightweight, mechanically adaptive applications. Such substantial density reduction is particularly advantageous for wearable electronics and soft robotic systems, as it enhances wearer comfort, mechanical compliance, and energy efficiency during prolonged or dynamic usage.

Notably, this structural transformation, enabled by the dynamic crosslinked network and its strong CO_2_ affinity, significantly surpasses conventional polymer foams processing methods, typically requiring harsh conditions (> 100 °C, > 10 MPa) [42, 68]. To explore the underlying mechanisms, first-principles simulations were conducted using VASP to quantitatively evaluate the interactions between the composite components and CO_2_ at the molecular scale, as shown in Fig. 2e-g. The results reveal that the binding energy of the dynamic disulfide bonds within poly-LA is significantly higher compared to that of the hydrogen bonds between carboxyl groups, carboxyl and imino groups, and the interactions between LA molecules and the IL (Fig. 2e). This dynamic crosslinked network is critical in providing the engineered composite with its mechanical stability and elasticity under various conditions. Interestingly, the introduction of CO_2_ during the structural transformation process reduces the binding energy and increases the bond lengths of these dynamic bonds, leading to a temporary weakening of intermolecular forces within the composite (Fig. 2e, f). This behavior facilitates enhanced molecular mobility within the matrix, which is essential for enabling structural reorganization and the formation of the microporous architecture.

The reduction in binding energy directly manifests the local plasticization effect induced by CO_2_, providing the molecular-level basis for the enhanced chain mobility. CO_2_ acts as a molecular plasticizer, lowering the energy barrier for polymer chain rearrangement, thereby promoting matrix expansion under mild conditions. Components within the engineered composite, particularly the ionic liquid, exhibit strong interactions with CO_2_ through hydrogen bonding and electrostatic forces, as shown in Fig. 2g. This strong affinity enhances CO_2_ solubility within the composite, even at low pressures, enabling efficient molecular reconfiguration and micropore formation [42, 68]. The SiO_2_-LA nanoparticles further contribute by serving as nanoscale sites for structural initiation, reducing the energy barrier for pore nucleation and stabilizing the newly formed microporous structure. These interrelated factors, including dynamic disulfide bonds, ionic liquid (IL)-CO_2_ interactions, and nanoparticle-induced heterogeneous nucleation, synergistically drive the formation of a well-defined microporous structure at room temperature and low pressure (2.0 MPa), a capability that is unattainable with conventional polymers such as polyolefins.

Additionally, we systematically evaluated the influence of processing parameters and composite composition on microporous development. As shown in Fig. S9, at lower CO_2_ pressure (1 MPa), limited pore formation occurred because the thermodynamic driving force was insufficient to overcome the nucleation energy barrier, thereby restricting bubble nucleation and growth, whereas at higher pressure (5 MPa), a finer and denser microporous architecture was obtained. This transition is attributed to the enhanced heterogeneous nucleation efficiency under elevated pressure, where reduced energy barriers at nanoparticle/polymer interfaces promote site-specific pore nucleation [42, 69]. Particle size analyses revealed that smaller nanoparticles provided increased heterogeneous nucleation sites, resulting in finer pore structures and higher pore densities (Fig. S10). Conversely, increasing nanoparticle loading elevated matrix rigidity through enhanced crosslinking, thus restricting structural expansion and leading to reduce pore sizes and overall porosity (Fig. S11). Composites lacking nanoparticles formed irregular and unstable micropores due to insufficient structural stabilization (Fig. S7). Additionally, an ionic liquid concentration of approximately 20 wt% was identified as optimal, effectively balancing molecular mobility and matrix stabilization to achieve the finest and densest pore architecture (Fig. S12). FTIR spectroscopy (Fig. S13) confirmed consistent chemical composition before and after microporous structuring. Based on these results, the composite formulation with 1 wt% SiO_2_-LA nanoparticles (20 nm) and 20 wt% IL, processed at 2.0 MPa CO_2_, was selected for subsequent evaluations owing to its well-defined microporous structure, optimal mechanical performance, and mild fabrication conditions.

### Mechanical Performance

**Fig. 3:**
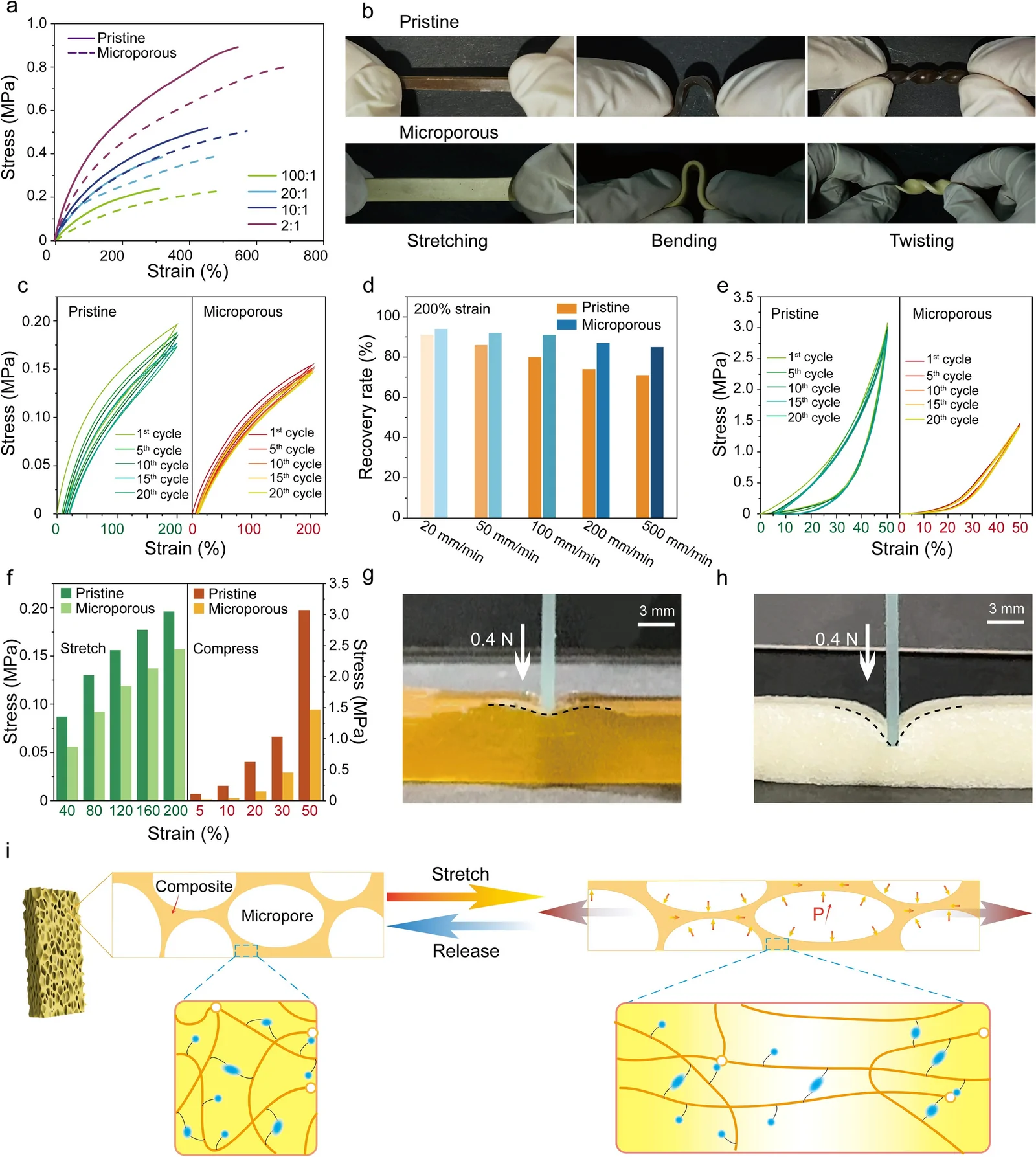
Robust mechanical properties and adaptability. **a** Stress–strain curves of the engineered composites with varying amounts of SiO_2_-LA, in both microporous and dense states. **b** Photographs showing the flexibility of the composite before and after microporous structuring. Comparison of **c** tensile cyclic stress–strain curves, **d** strain recovery rates at 200% deformation under different tensile rates, and **e** compressive cyclic stress–strain curves for the microporous and dense composite. **f** Comparison of required stress for equivalent tensile/compressive strain in microporous versus dense composites. Adaptive deformation of **g** dense and **h** microporous composites under gentle compressive stress. **i** Schematic illustration of elastic recovery in the microporous composite

The introduction of a microporous architecture markedly enhances the mechanical performance and adaptability of the composite elastomer. Compared to its dense counterpart, the microporous composite demonstrates significantly improved elongation at break (Figs. 3a and S14), attributed to efficient stress dispersion and reduced local stress concentrations within its cellular structure. At the same time, the reduced tensile strength counterbalances the increased strain, leading to an overall toughness comparable to that of the dense composite (Fig. S14), indicating that the microporous design enhances deformability while preserving toughness. Both porous and dense samples display excellent flexibility, as illustrated in Fig. 3b. Notably, the incorporation of porosity maintains structural integrity comparable to the dense state, while imparting superior elastic behavior. During cyclic tensile tests, the microporous composite exhibits substantially reduced hysteresis (Fig. 3c), indicating enhanced elastic resilience and minimized energy dissipation. Specifically, the elastic recovery rate increases from 85% in the dense state to approximately 93% post-structuring, consistently exceeding 90% even at high strain rates (Fig. 3d), underscoring its robust dynamic response. Under cyclic compression, the microporous elastomer displays exceptional rebound characteristics, characterized by significantly narrowed hysteresis loops (Fig. 3e).

Based on the measured data, we calculated the dissipation energy and dissipation efficiency for each cycle under both tensile and compressive loading (Fig. S15). Compared with the dense counterpart, the microporous elastomer exhibited markedly lower dissipation energy and reduced dissipation efficiency, while maintaining substantially superior cyclic stability, indicative of enhanced elastic resilience under repeated loading. Furthermore, it retains over 80% of its initial stress after 500 tensile cycles at 100% strain and compression cycles at 50% strain (Fig. S16), confirming its long-term mechanical stability. The enhanced resilience originates primarily from the unique interplay between the microscale cellular framework and the increased polymer chain mobility afforded by expanded free volume (Figs. 3i and S17) [70]. During tensile or compressive deformation, the polymer cell walls store elastic potential energy, while deformation of the closed-cell structures increases the internal gas pressure. Upon unloading, the re-equilibration of this internal pressure acts in concert with the elastic recoil of the cell walls, producing a micro-spring effect that markedly amplifies the macroscopic rebound. Moreover, the microporous architecture increases free volume, thereby reducing interchain frictional dissipation, while simultaneously blunting crack tips and lowering the fracture energy release rate. Collectively, these effects account for the reduced energy losses and lower dissipation efficiency observed in the microporous elastomer.

The microporous structure also substantially elevates mechanical adaptability, *i.e.*, the capacity to accommodate mechanical and shape changes, as evidenced by the requirement of considerably lower stress to achieve identical strain levels, as well as the ability to accommodate larger deformation under equivalent stress conditions compared to the dense composite (Fig. 3f-h).This advantageous property results directly from the microporous network’s ability to accommodate mechanical deformation through localized strain zones and effectively dissipate applied forces (Figs. 3i and S17). Additionally, the enhanced free volume within the porous architecture significantly improves polymer chain mobility, allowing the material to conform readily and sensitively to complex shapes and dynamic mechanical environments. Consequently, the composite elastomer can easily adapt to intricate deformation patterns, ideal for adaptive interfaces and responsive components in flexible devices. Notably, the microporous composite demonstrates remarkable structural stability. As illustrated in Fig. S18, the tensile and compressive cyclic performances remain stable, with no noticeable degradation after exposure to ambient conditions for 7, 15, 30, and 60 days.

The ultralight nature of the microporous elastomer substantially reduces mechanical impedance, minimizing resistance to motion, and significantly enhancing comfort during prolonged use. The synergistic integration of lightweight structure and enhanced mechanical adaptability highlights the exceptional potential of this microporous elastomeric design for advanced wearable electronics, soft robotics, and human–machine interfaces, where precise mechanical responsiveness and sustained user comfort are essential.

### Sensing Performance

**Fig. 4:**
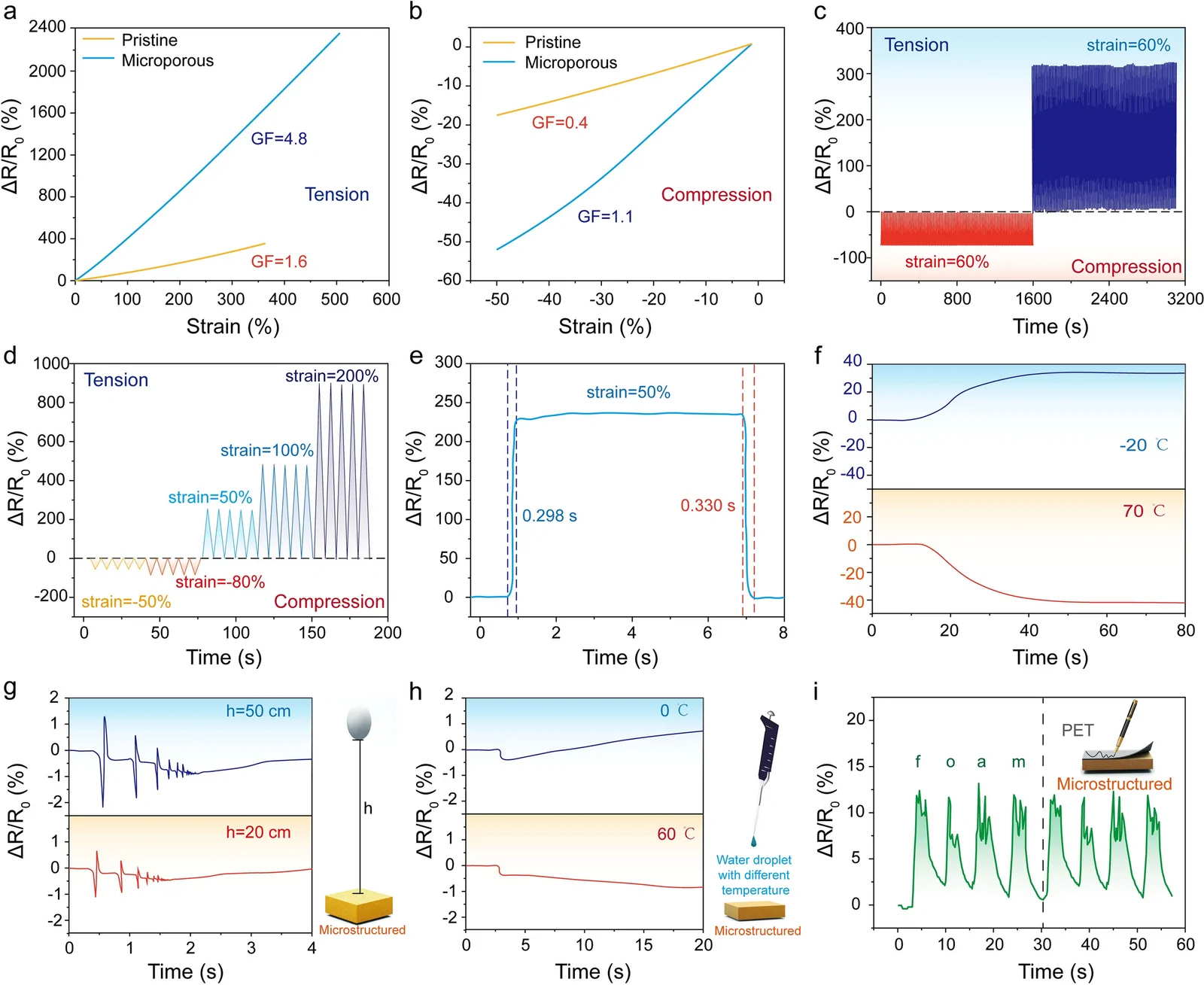
Highly sensitive and stable real-time sensing capability. Variation in relative resistance under **a** tensile stress and **b** compressive stress. **c** Stability over 100 cycles of tensile and compressive stress. **d** Performance at different strain levels. **e** Instantaneous response of the electrical signal to deformation. **f** Sensitivity to different temperatures. **g** Response to a ping-pong ball dropped from varying heights. **h** Reaction to water droplets of varying temperatures. **i** Response during handwriting of “foam” on the surface

Leveraging its optimized mechanical properties, structural flexibility, and intrinsic ionic conductivity, the porous-structured composite exhibits exceptional sensing performance, characterized by enhanced sensitivity, rapid responsiveness, and excellent signal stability [71, 72]. To elucidate the influence of pore architecture on conductivity, bulk ionic conductivity was measured across a range of foaming pressures (Fig. S19) and correlated with pore statistics derived from SEM image analysis (pore size, pore density, and porosity). The conductivity decreases monotonically with increasing foaming pressure, consistent with the formation of smaller pores and higher pore densities/porosity, which reconstruct the conductive network by reducing the number of effective pathways per unit volume, increasing tortuosity, and introducing pore-wall interfacial resistance. Nevertheless, long-range conduction remains percolated, with the composite retaining ~ 65% of its dense-state conductivity under a 2 MPa foaming condition. Notably, introducing microcellular architecture notably increases the composite’s sensitivity to tensile and compressive strains, achieving enhancements of approximately 3.0 and 2.75 times, respectively, compared to its dense-state counterpart (Fig. 4a, b). Such improved sensitivity arises primarily from the interconnected microcellular structure, which effectively amplifies localized deformation, thus enabling greater strain under equivalent external stress. Moreover, repeated contact-separation events among internal pore walls significantly contribute to enhanced strain-sensing signals. The dynamic change in electrical resistance under tensile strain is mechanistically linked to the amplified deformation: stretching separates conductive pore walls and elongates/narrows ionic transport pathways within the polymer matrix, leading to an increase in overall resistance. In contrast, compressive strain forces pore walls into closer contact, shortening and widening conductive pathways and thereby reducing resistance. Stability tests further emphasize the composite’s reliability in dynamic environments. Under cyclic tensile and compressive loading (Fig. 4c, d), the porous-structured composite maintains stable and repeatable electrical outputs without performance deterioration over 100 cycles. This robustness is attributed to its dynamic covalent network, enabling rapid reversible interactions that sustain mechanical integrity and consistent electrical pathways upon repeated deformation. Specifically, the dynamic network allows for local reconfiguration and healing at stress-concentrated points (*e.g.*, pore junctions), mitigating the risk of permanent damage or crack propagation that could disrupt conductive paths. In addition, the structural compression inherent in each cycle promotes intimate contact between pore walls, facilitating the re-establishment of efficient conductive pathways. Furthermore, the interconnected microporous architecture effectively distributes stress, minimizing local failures and preserving signal fidelity under prolonged dynamic conditions.

Notably, the composite demonstrates remarkable real-time responsiveness. Upon rapid stretching to 50% strain within 0.3 s, its relative resistance changes instantaneously, returning promptly and precisely to the initial state upon stress removal (Fig. 4e). Such rapid recovery is enabled by the synergistic combination of dynamic crosslinking—facilitating quick reformation of polymer chains—and the porous structure, which expedites elastic rebound by efficient stress dissipation. Additionally, continuous ionic pathways supported by hydrogen bonding and electrostatic interactions ensure immediate recovery of conductivity, underpinning accurate and stable real-time strain monitoring capabilities. Apart from mechanical sensing, the porous-structured composite shows high sensitivity to temperature fluctuations (Fig. 4f), responding distinctly through changes in ionic conductivity within its internal architecture. Strikingly, the material precisely captures subtle mechanical stimuli, such as low-impact events from a ping-pong ball dropped at varying heights (Fig. 4g), an ability absent in its dense counterpart (Fig. S20). Moreover, even after repeated cyclic loading at a small strain amplitude of 1%, the sensing signals remain real time and stable without noticeable drift (Fig. S21), demonstrating reliable responsiveness to subtle mechanical stimuli. Furthermore, the composite sensitively responds to water droplets, where the abrupt signal change originates from transient deformation induced by droplet impact, followed by a gradual variation arising from temperature effects (Fig. 4h). The latter behavior is attributed to the temperature dependence of ionic mobility, whereby rising temperature accelerates ion migration and consequently increases the electrical conductivity of the composite (Fig. S22). Such multifaceted sensing performance highlights its suitability for delicate real-time monitoring scenarios.

The porous-structured composite also demonstrates excellent capability in capturing intricate signals, as illustrated by simulated handwriting experiments (Fig. 4i), clearly distinguishing individual strokes and letters through characteristic electrical responses. Such performance underscores the composite’s potential for advanced interactive interfaces and wearable electronics. Collectively, these superior sensing attributes arise from its engineered porous architecture, significantly amplifying localized strain signals and enabling rapid, precise electromechanical feedback—thereby establishing it as a highly promising candidate for advanced sensing and soft electronics applications.

### Room Temperature Self-Healing

Self-healing capability is crucial for maintaining long-term reliability, as materials in practical applications frequently encounter mechanical damage and performance deterioration [73]. Unlike conventional materials with rigid, non-repairable structures, the engineered porous-structured composite integrates dynamic disulfide and hydrogen bonds, enabling intrinsic self-healing at room temperature without external stimuli.

**Fig. 5:**
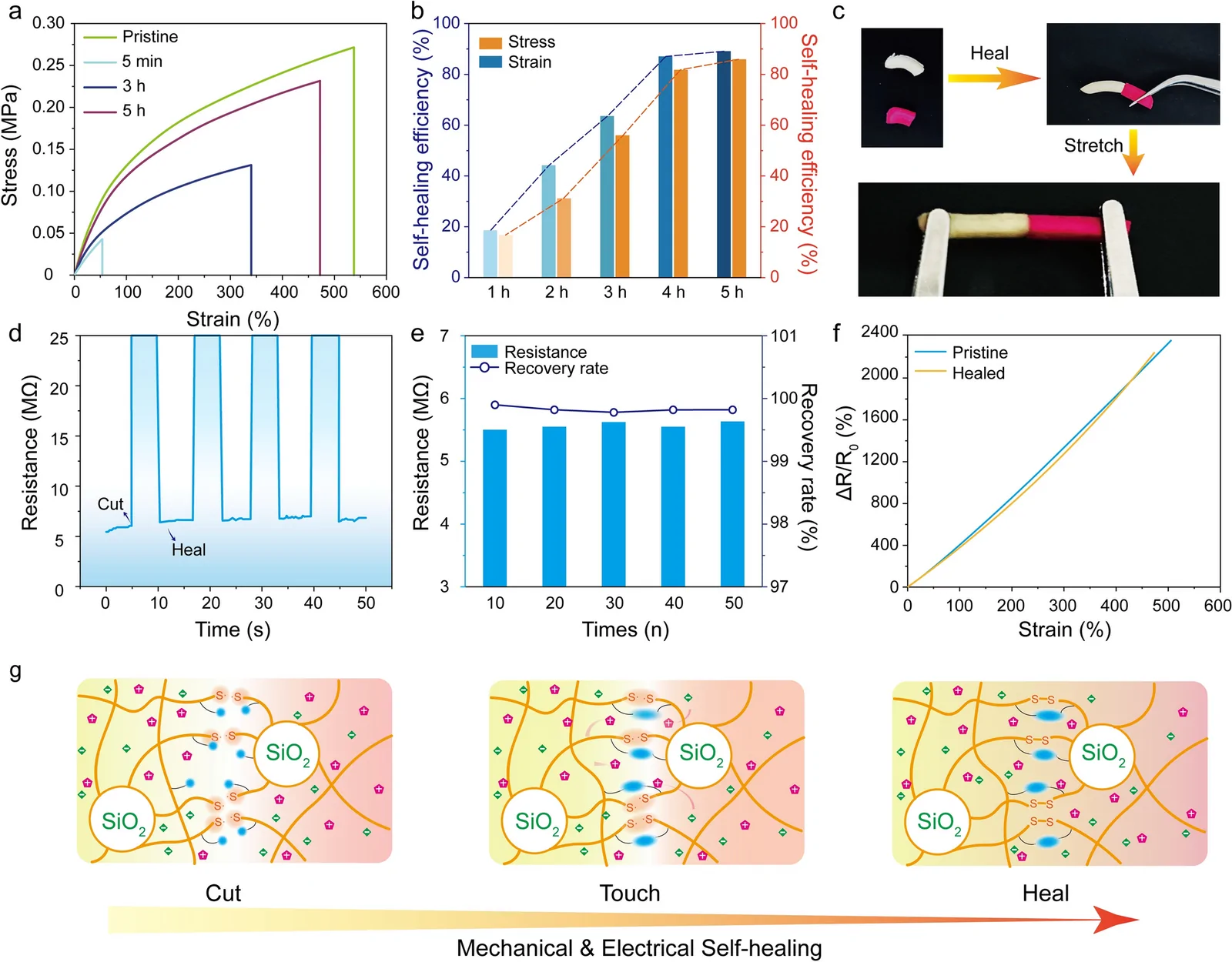
Efficient self-healing capability at room temperature. **a** Stress–strain curves at different self-healing intervals. **b** Self-healing efficiency for tensile stress and strain over time. **c** Photographic sequence of the self-healing process. **d** Recovery of electrical resistance after cutting. **e** Changes in resistance and recovery rate during repeated cutting and reconnection cycles. **f** Comparison of gauge factor before and after self-healing. **g** Schematic diagram illustrating the self-healing mechanism for both mechanical and electrical properties in the microporous structured composite

Mechanical and electrical self-healing performances were systematically evaluated. As shown in Fig. 5a, b, the porous-structured composite recovered approximately 90% of its original stress and strain within 5 h under ambient conditions. Photographic evidence (Fig. 5c) clearly demonstrates its exceptional self-repair capability upon simple recontact after cutting. This accelerated self-healing is primarily attributed to the interconnected porous architecture, which significantly increases interfacial contact areas and polymer chain mobility, facilitating rapid reformation of dynamic chemical bonds at damage sites. Meanwhile, the self-healing performance exhibits a pronounced dependence on temperature and humidity. As shown in Fig. S23, at low temperature (0 °C), restricted chain mobility leads to reduced healing efficiency and slower healing rate, although the material still retains ~ 60% of its healing efficiency. In contrast, elevated temperatures accelerate chain mobility, resulting in enhanced healing efficiency and faster recovery. In terms of humidity (Fig. S24), moderate moisture content increases the free volume of the polymer network at the fractured interface, yielding an optimal healing efficiency at ~ 60% relative humidity. However, further increases in humidity introduce excessive water molecules that competitively occupy hydrogen bonding sites, thereby diminishing the healing efficiency. Similarly, the composite exhibited remarkable electrical self-healing performance. As depicted in Fig. 5d, electrical conductivity was immediately restored upon rejoining the severed sections, even after multiple repeated cut-reconnection cycles. After four cycles, electrical resistance only slightly increased (from 5.03 to 5.11 MΩ), demonstrating outstanding conductivity recovery. Furthermore, after 50 consecutive damage-repair cycles, the composite retained approximately 98% of its original electrical conductivity (Fig. 5e). Importantly, repeated self-healing did not degrade the composite’s sensing performance, as reflected by stable gauge factor (GF) values and consistent electrical signals (Fig. 5f).

The underlying mechanism for the simultaneous mechanical and electrical self-healing performance is illustrated in Fig. 5g. Upon damage, ion transport pathways within the polymer network are disrupted, temporarily interrupting conductivity. However, the porous structure’s enhanced interfacial area and free volume significantly expedite the re-establishment of conductive pathways upon contact, rapidly recovering electrical functionality. Concurrently, dynamic hydrogen bonds initially form non-covalent interactions across the damaged interfaces, subsequently transitioning into stable covalent bonds via disulfide bond exchange. This synergy effectively restores mechanical integrity and elasticity, ensuring simultaneous recovery of both mechanical and electrical functionalities. Thus, the uniquely designed porous-structured composite demonstrates outstanding durability and reliability, providing a robust strategy for fabricating self-healing conductive elastomers suitable for advanced sensing and flexible electronic applications.

### Recyclability and Performance Advantages

**Fig. 6:**
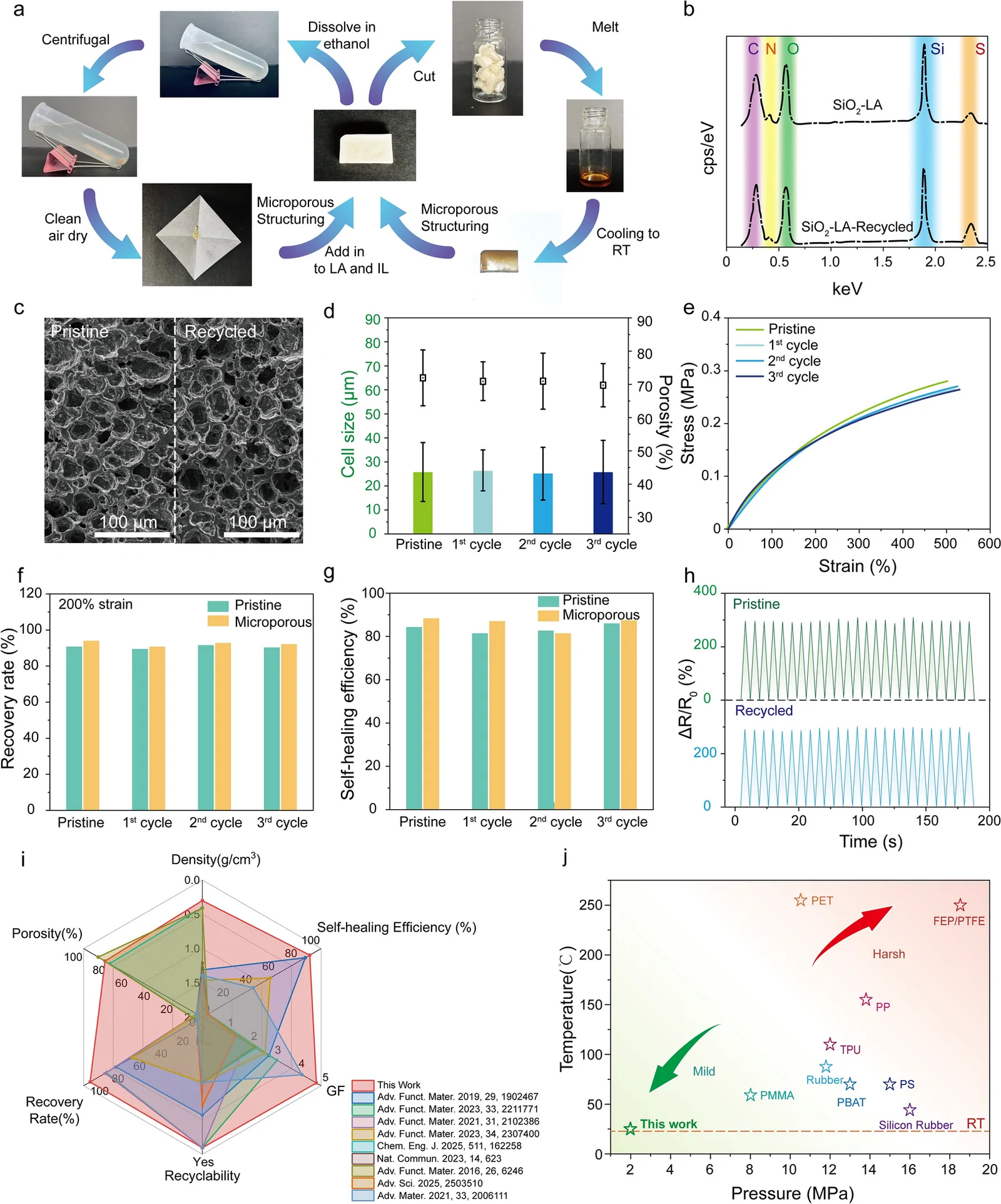
Full recyclability and superior performance. **a** Schematic of the closed-loop recycling process for the engineered composite. **b** EDS spectra of SiO_2_-LA nanoparticles before and after recycling. **c** SEM images of microporous morphology before and after three recycling cycles. **d** Evolution of pore size and porosity with increasing recycling cycles. **e** Stress–strain behavior of the microporous composite pre- and post-recycling. **f** Strain recovery ratio at 200% deformation. **g** Self-healing efficiency of microporous and dense composites over multiple cycles. **h** Comparison of sensing performance before and after recycling. **i** Performance comparison with peer-reported elastic conductive composites. **j** Comparison of processing conditions between the engineered elastomer and conventional polymer systems, highlighting a > 80% reduction in both temperature and pressure

As illustrated in Fig. 6a, the engineered porous-structured composite demonstrates outstanding recyclability, enabled by its dynamic crosslinked molecular network. The composite can be readily depolymerized in ethanol through the cleavage of dynamic disulfide, hydrogen bonds, and electrostatic interactions, facilitating straightforward separation and recovery of pristine SiO_2_-LA nanoparticles, ionic liquids (IL), and polymer precursors. After centrifugation, SiO_2_-LA nanoparticles exhibit a recovery rate exceeding 99%, even after five consecutive recycling cycles (Fig. S25). FTIR and EDS analyses (Figs. 6b and S26) confirmed no significant changes in the chemical structure or elemental composition after recycling, validating the feasibility of the recycling process and the material’s chemical stability [74].

Notably, the recycled composite maintains consistent morphology, mechanical robustness, and functional performance across multiple reuse cycles. SEM characterization (Fig. 6c, d) reveals negligible changes in pore size and porosity after repeated recycling, indicating excellent structural preservation. Moreover, assessments of mechanical properties, elastic resilience, self-healing and sensing performance (Fig. 6e-h) show that the composite consistently retains properties comparable to its original state. This outstanding stability arises from the reversible nature of dynamic chemical interactions, allowing repeated structural reconstruction without performance degradation. Overall, the developed composite presents significant advantages in recyclability, property stability, and sustainable reuse, offering considerable potential as an eco-friendly, high-performance alternative for green manufacturing and advanced polymer-based functional materials.

Additionally, the engineered conductive elastomer exhibits distinct performance advantages over previously reported systems, especially regarding low density, electromechanical sensitivity, self-healing efficiency, and elastic resilience (Fig. 6i and Table S1). The introduction of a well-defined microporous structure uniquely imparts simultaneous ultralight weight and exceptional mechanical adaptability, traits rarely achieved by traditional biomass-based conductive elastomers. Compared to porous conductive materials like aerogels, which typically suffer from brittleness and limited deformability restricting them to compressive sensing applications, this elastomer demonstrates robust tensile stretchability and mechanical responsiveness ideal for wearable and flexible electronics. Critically, the microporous structure is achieved under mild conditions (2.0 MPa CO_2_ at room temperature), circumventing the elevated temperatures (> 100 °C), high pressures (> 10 MPa), and harsh solvents typically required by conventional foaming or templating methods (Fig. 6j). Enabled by the CO_2_-affinitive dynamic network, this environmentally friendly and energy-efficient fabrication significantly enhances scalability and sustainability. Collectively, these attributes underscore the material’s significant potential for advanced soft electronics, including wearable sensing, soft robotics, and adaptive human–machine interfaces.

## Conclusion

In summary, we demonstrate a rational molecular design strategy to fabricate biomass-derived conductive elastomers featuring a highly adaptive microporous structure via a mild, room temperature process. Distinct from traditional elastomers and porous materials, this design simultaneously attains exceptional lightweighting, superior micromechanical sensitivity, and remarkable mechanical compliance and resilience. The intrinsic microporous architecture imparts a characteristic micro-spring effect, significantly enhancing the elastomer’s ability to dissipate stress, achieve rapid recovery from deformation, and deliver precise real-time electrical feedback under mechanical stimuli ranging from < 1% to > 200% strain. Moreover, the embedded dynamic crosslinking network imparts robust room temperature self-healing capability and outstanding recyclability, addressing critical sustainability and durability challenges in flexible electronics. The molecular insights obtained from first-principles simulations clarify fundamental relationships between the elastomer’s dynamic bonding and micropore formation mechanisms, providing a theoretical basis for enhanced functionalities. By combining facile, scalable fabrication with exceptional performance, this elastomeric platform presents significant opportunities for the advancement of sustainable soft electronics, opening new pathways toward intelligent wearable sensors, adaptive human–machine interfaces, and next-generation soft robotics.

## Supplementary Information

Below is the link to the electronic supplementary material.

Supplementary file1 (DOCX 18630 KB)
