# Supplementary material for: Dynamic Network- and Microcellular Architecture-Driven Biomass Elastomer toward Sustainable and Versatile Soft Electronics
Source: Nanomicro Lett. 2025 Dec 13;18:88. doi: 10.1007/s40820-025-01942-7 (PMC12701213; doi:10.1007/s40820-025-01942-7)
Supplement: Supplementary file 1 — Supplementary file1 (DOCX 18630 KB) [file 40820_2025_1942_MOESM1_ESM.docx]

Supporting Information for

**Dynamic Network- and Microcellular Architecture-Driven Biomass Elastomer toward Sustainable and Versatile Soft Electronics**

Shanqiu Liu^1,#,^*, Yi Shen^1,#^, Yizhen Li^2^, Yunjie Mo^1^, Enze Yu^1^, Taotao Ge^1^, Ping Li^3^*, Jingguo Li^2^*

^1^Institute for Frontiers and Interdisciplinary Science, Zhejiang University of Technology, Hangzhou 310014, P.R. China

^2^State Key Laboratory of Advanced Environmental Technology, Department of Environmental Science and Engineering, University of Science and Technology of China, Hefei 230026, P. R. China

^3^School of Materials Science and Engineering, Xi’an Jiaotong University, Xi’an 710049, P. R. China

^#^Shanqiu Liu and Yi Shen contributed equally to this work.

*Corresponding authors. E-mail: [shanqiuliu@zjut.edu.cn](mailto:shanqiuliu@zjut.edu.cn) (Shanqiu Liu); [pli@xjtu.edu.cn](mailto:pli@xjtu.edu.cn) (Ping Li); [lijg@ustc.edu.cn](mailto:lijg@ustc.edu.cn) (Jingguo Li)

**Supplementary Figures and Tables**


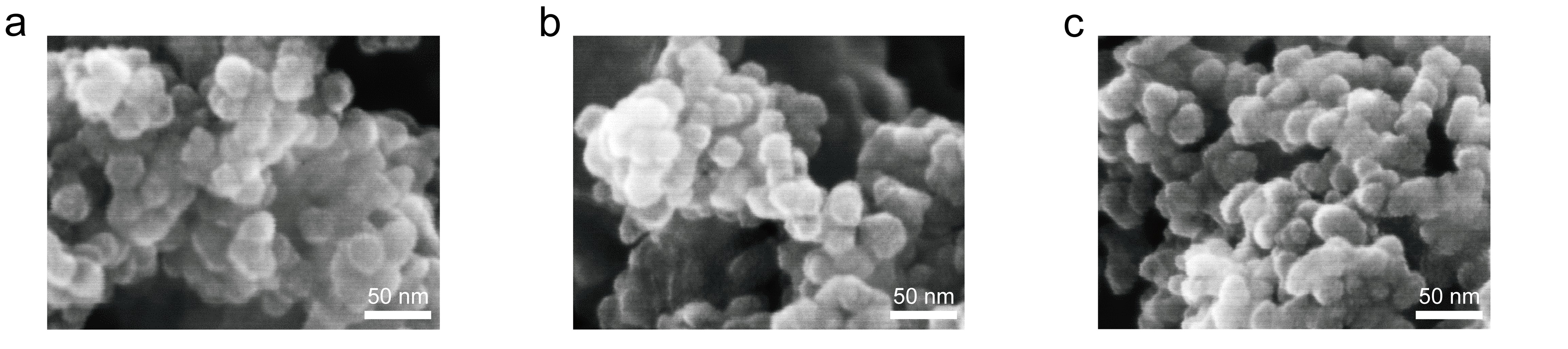


**Fig. S1** SEM images of **a** SiO_2_, **b** SiO_2_-NH_2_, and **c** SiO_2_-LA


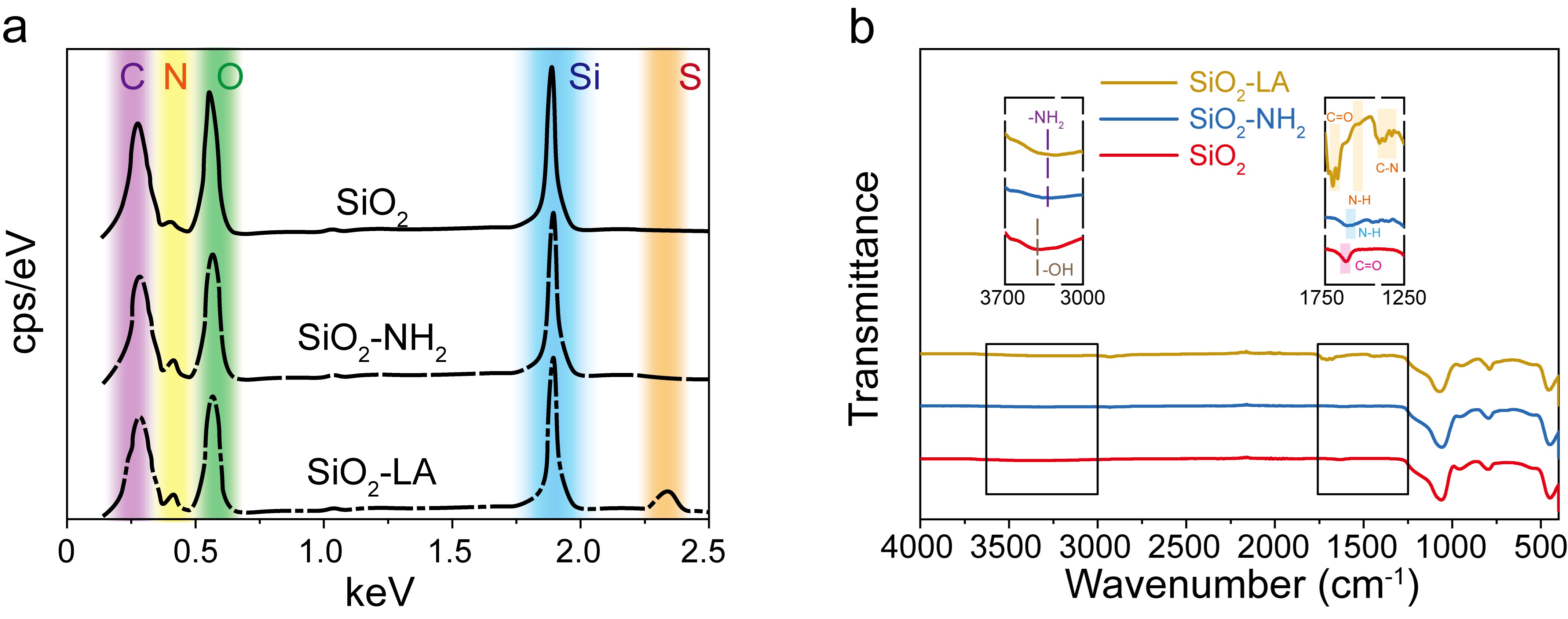


**Fig. S2** **a** EDX spectra and **b** FT-IR spectra of SiO_2_, SiO_2_-NH_2_ and SiO_2_-LA

**
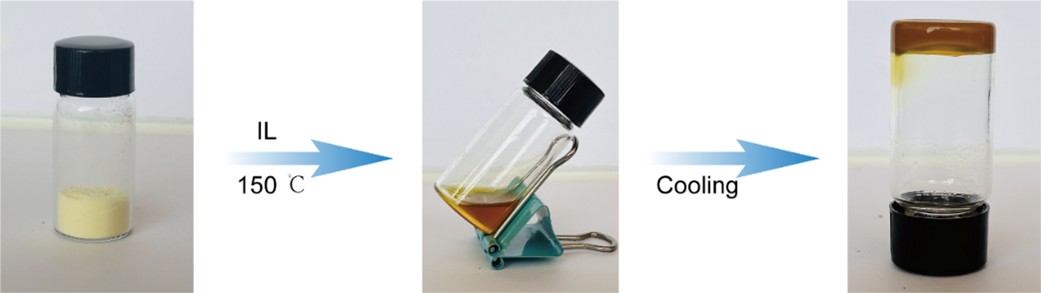
**

**Fig. S3** Digital photos for the preparation of the elastic composite


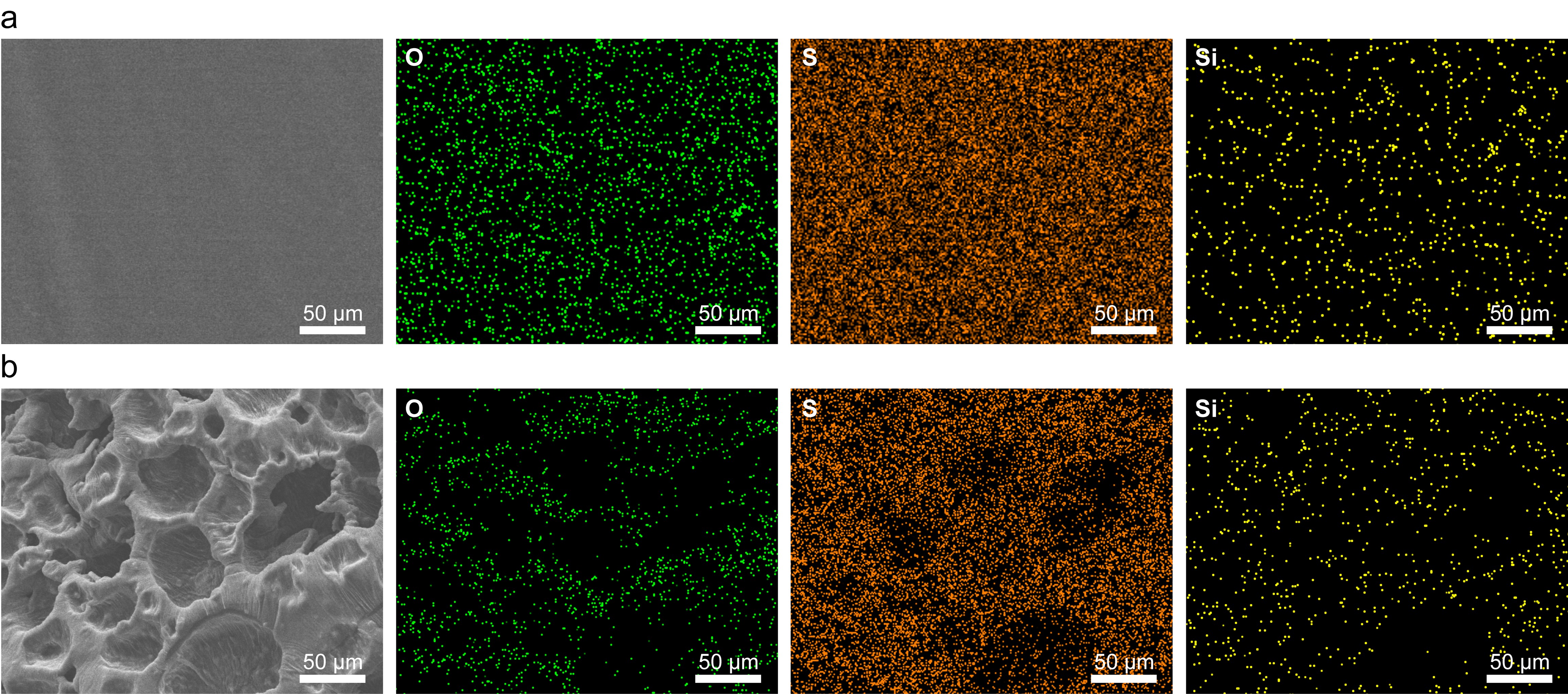


**Fig. S4** SEM and EDX spectra of composites containing 1 wt% 20 nm nanoparticles: **a** before and **b** after microporous structuring


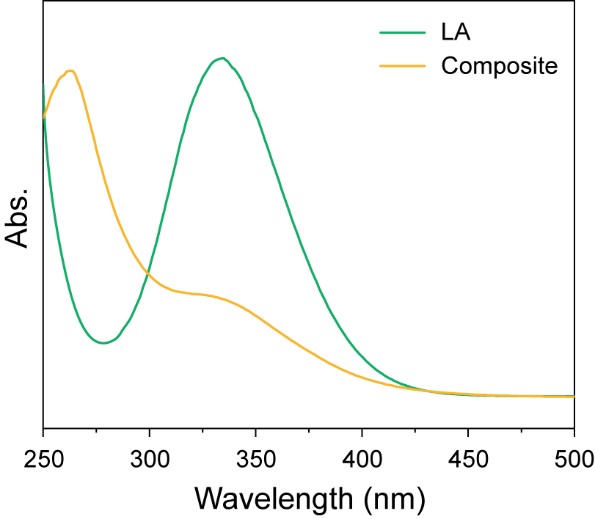


**Fig. S5** UV absorption spectra of lipoic acid and the elastic composite


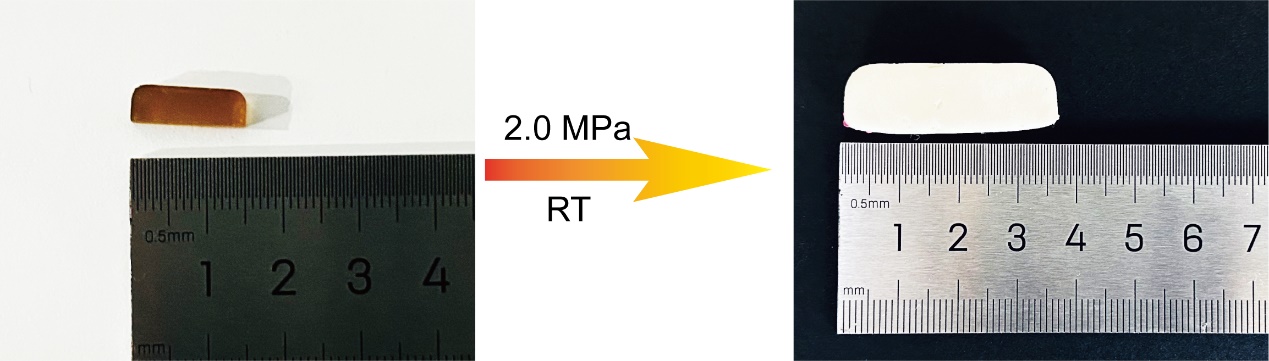


**Fig. S6** Digital photographs of the composite before and after microporous structuring


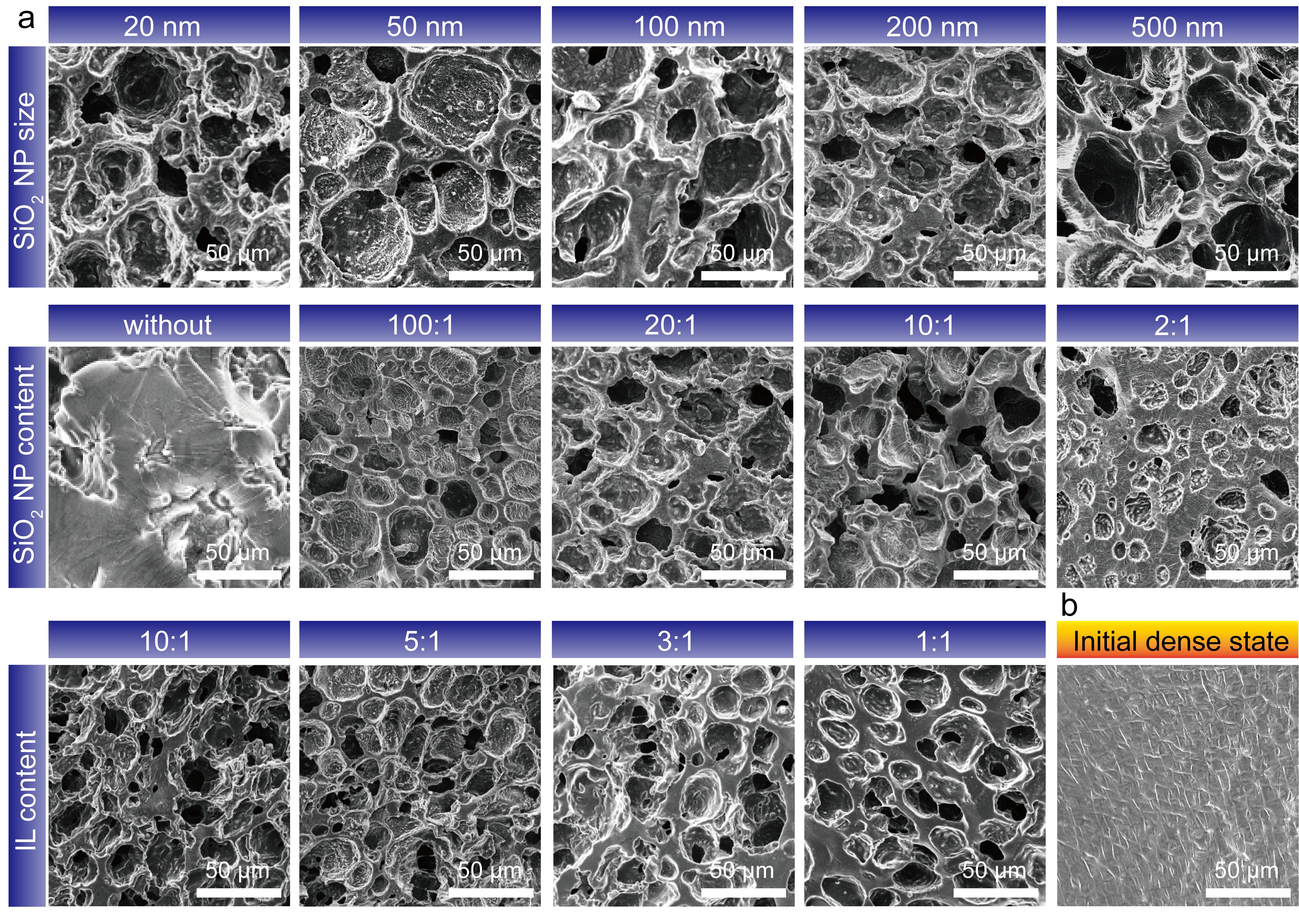


**Fig. S7** SEM images showing the microporous morphology of composites with different composition ratios. **a** After microporous structuring and **b** in the initial dense state


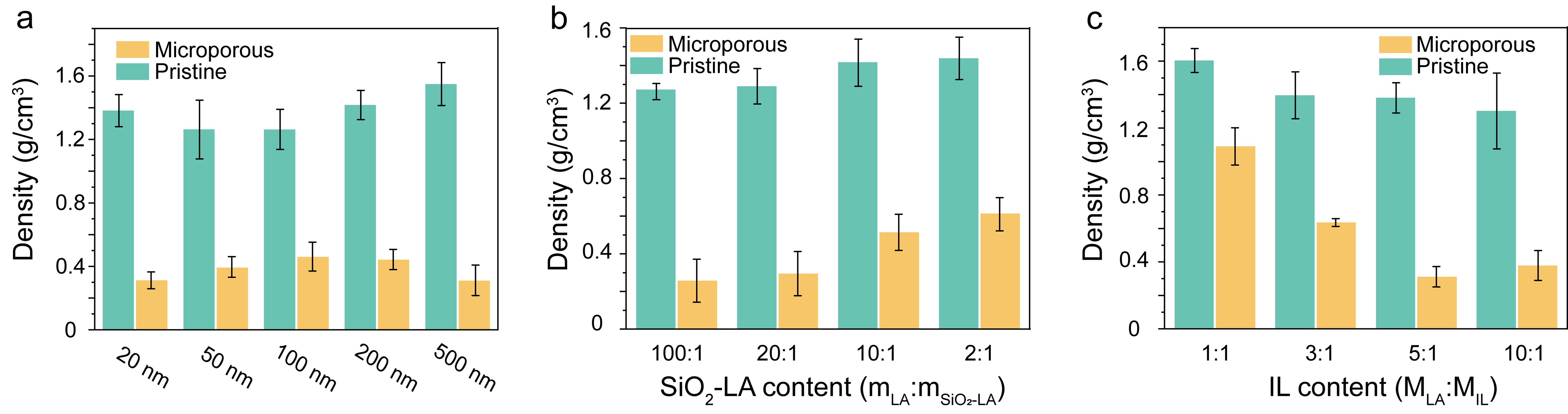


**Fig. S8** Density of composites with varying compositions before and after microporous structuring. **a** Composites containing 1 wt% nanoparticles of different sizes and 20 wt% ionic liquid. **b** Composites containing varying contents of 20 nm nanoparticles and 20 wt% ionic liquid. **c** Composites containing 1 wt% 20 nm nanoparticles and varying contents of ionic liquid


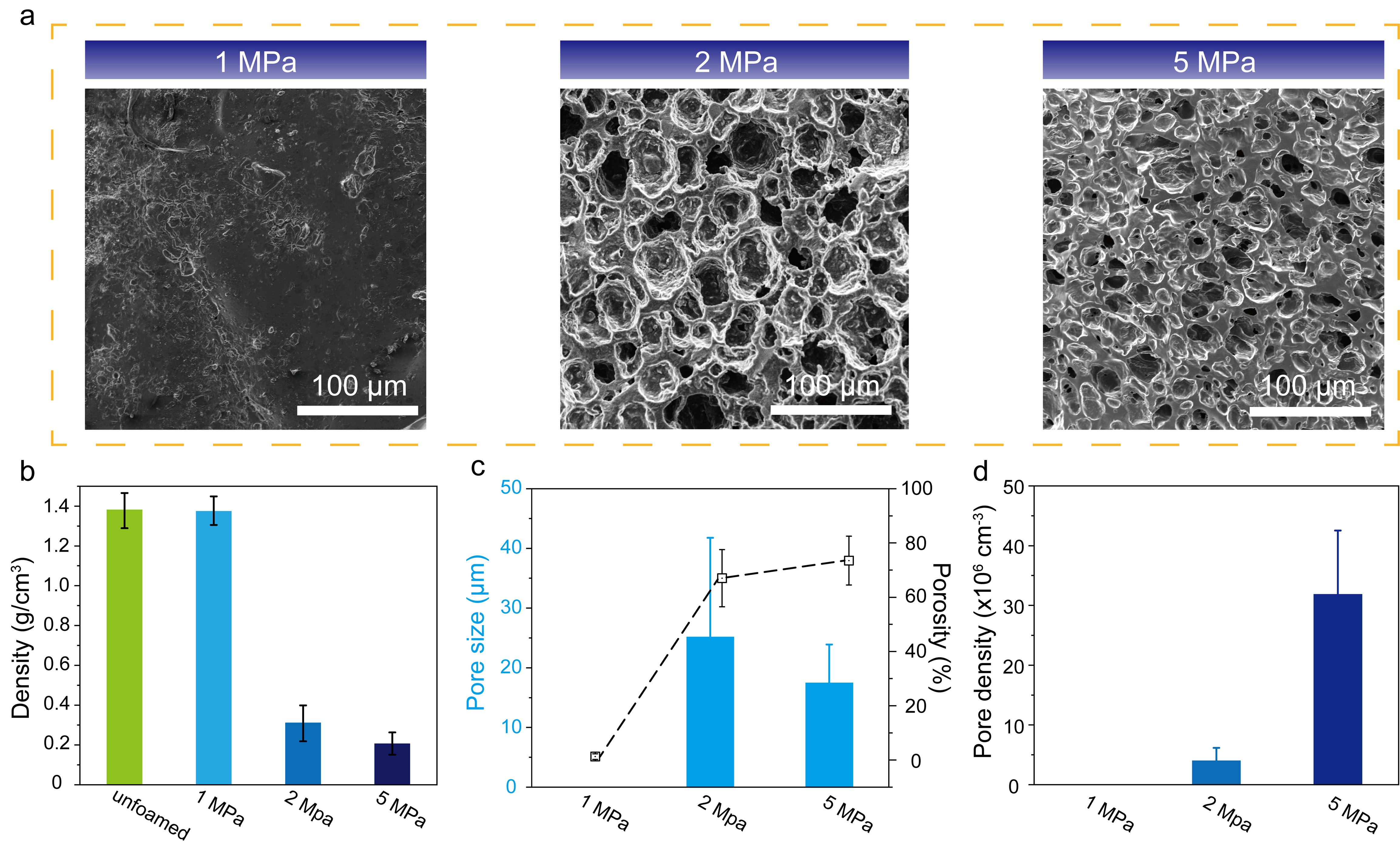


**Fig. S9** SEM images and structural parameters of composites with 1 wt% 20 nm nanoparticles and 20 wt% ionic liquid prepared under different CO₂ saturation pressures: **a** microporous morphology, **b** density, **c** average pore size and porosity, and **d** pore density


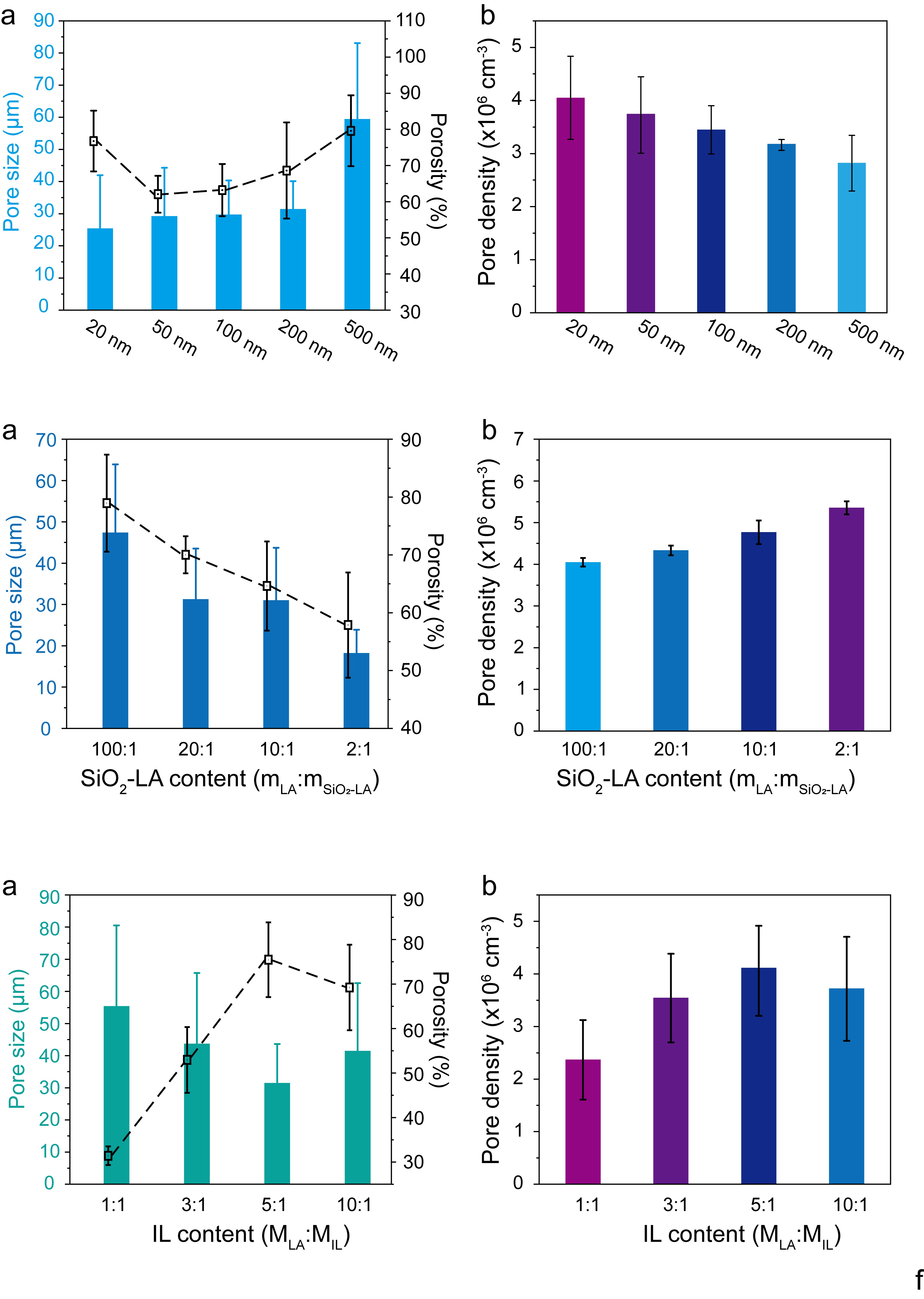


**Fig. S10** Microporous composites with 1 wt% SiO₂-LA and 20 wt% ionic liquid: **a** pore size and porosity, and **b** pore density as a function of nanoparticle size under CO_2_ saturation at 2.0 MPa


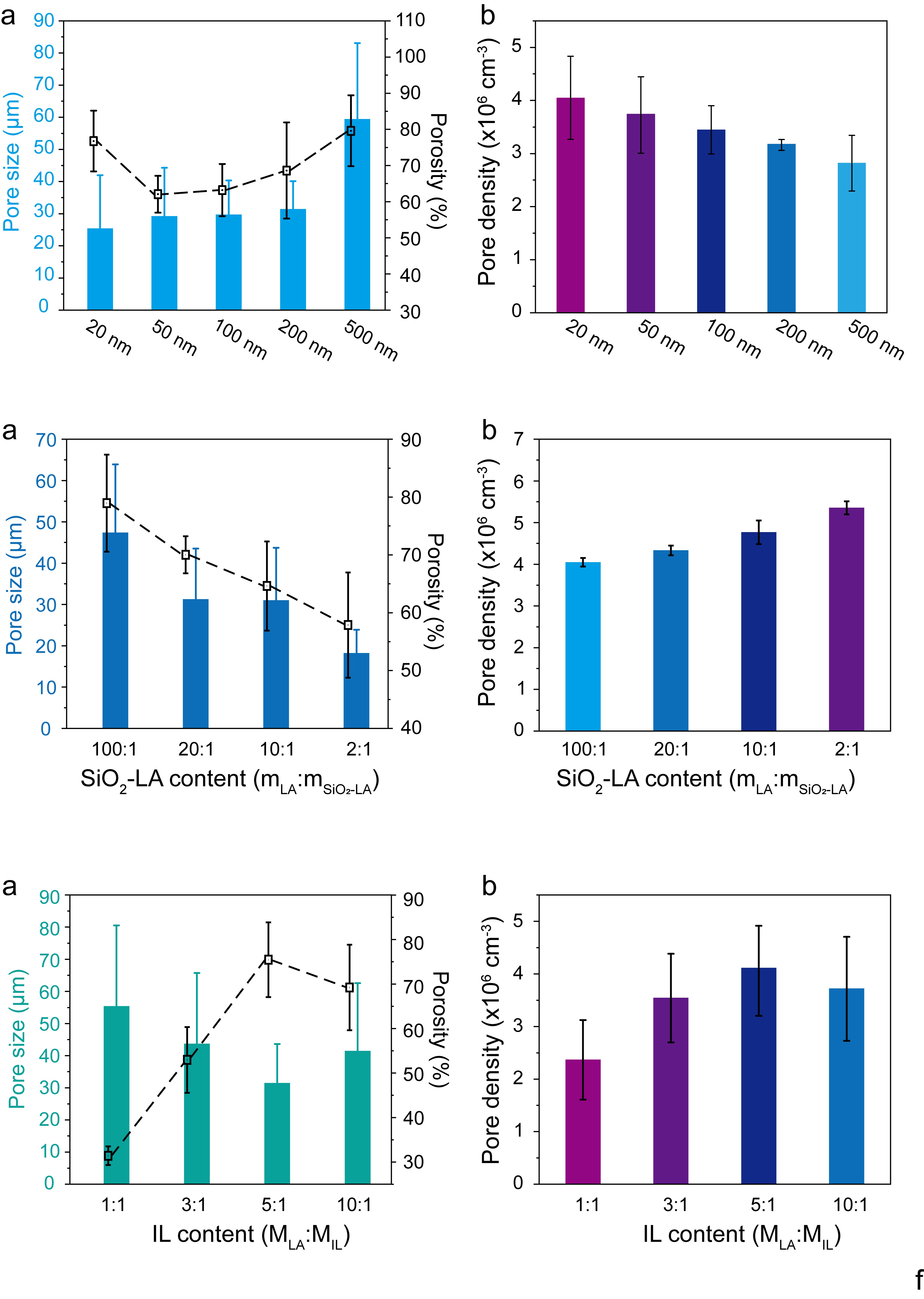


**Fig. S11** Microporous composites with 20 nm nanoparticles and 20 wt% ionic liquid: **a** pore size and porosity, and **b** pore density as a function of nanoparticle content under CO_2_ saturation at 2.0 MPa


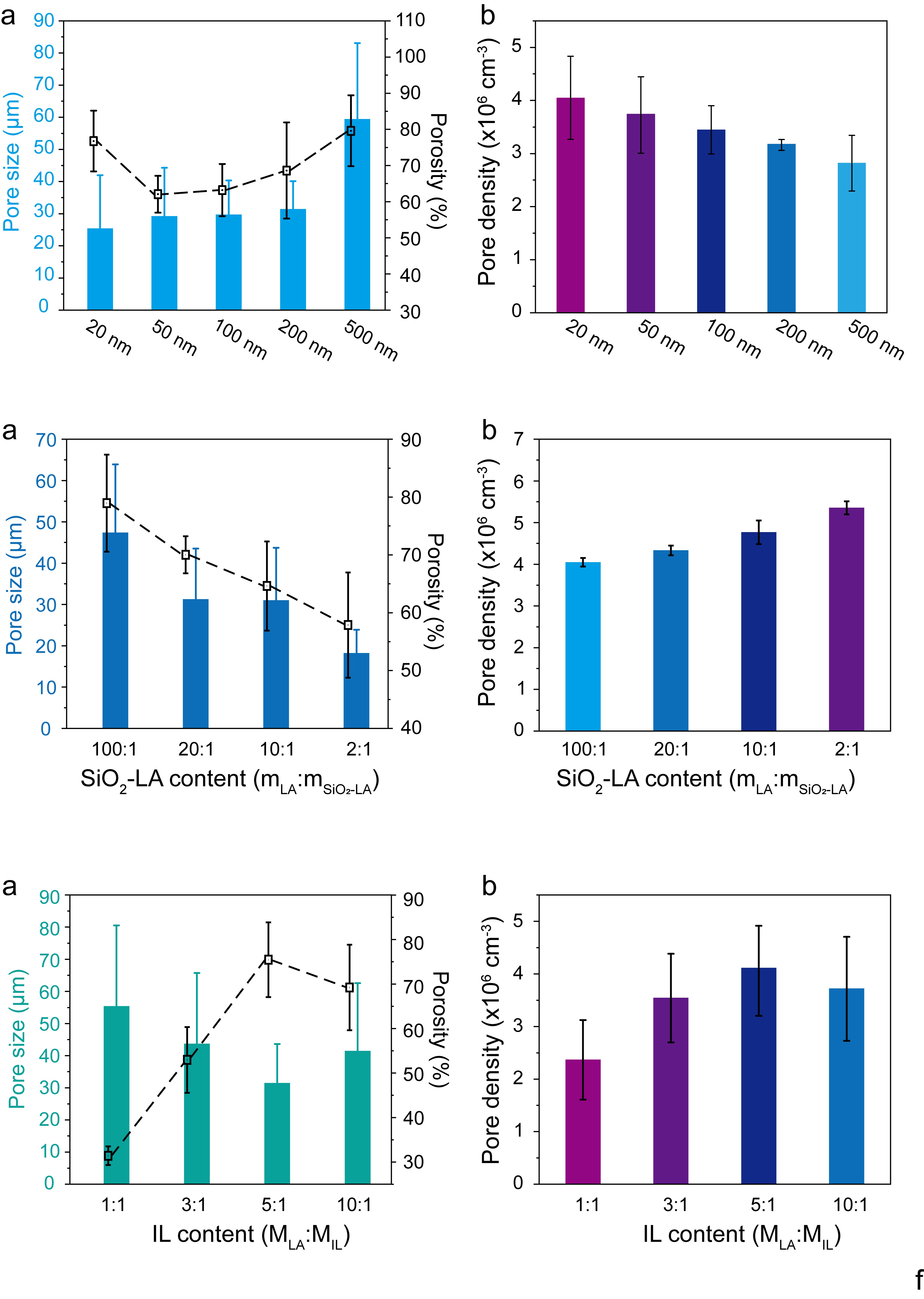


**Fig. S12** Microporous composites with 1 wt% 20 nm nanoparticles: **a** pore size and porosity, and **b** pore density as a function of ionic liquid content under CO_2_ saturation at 2.0 MPa


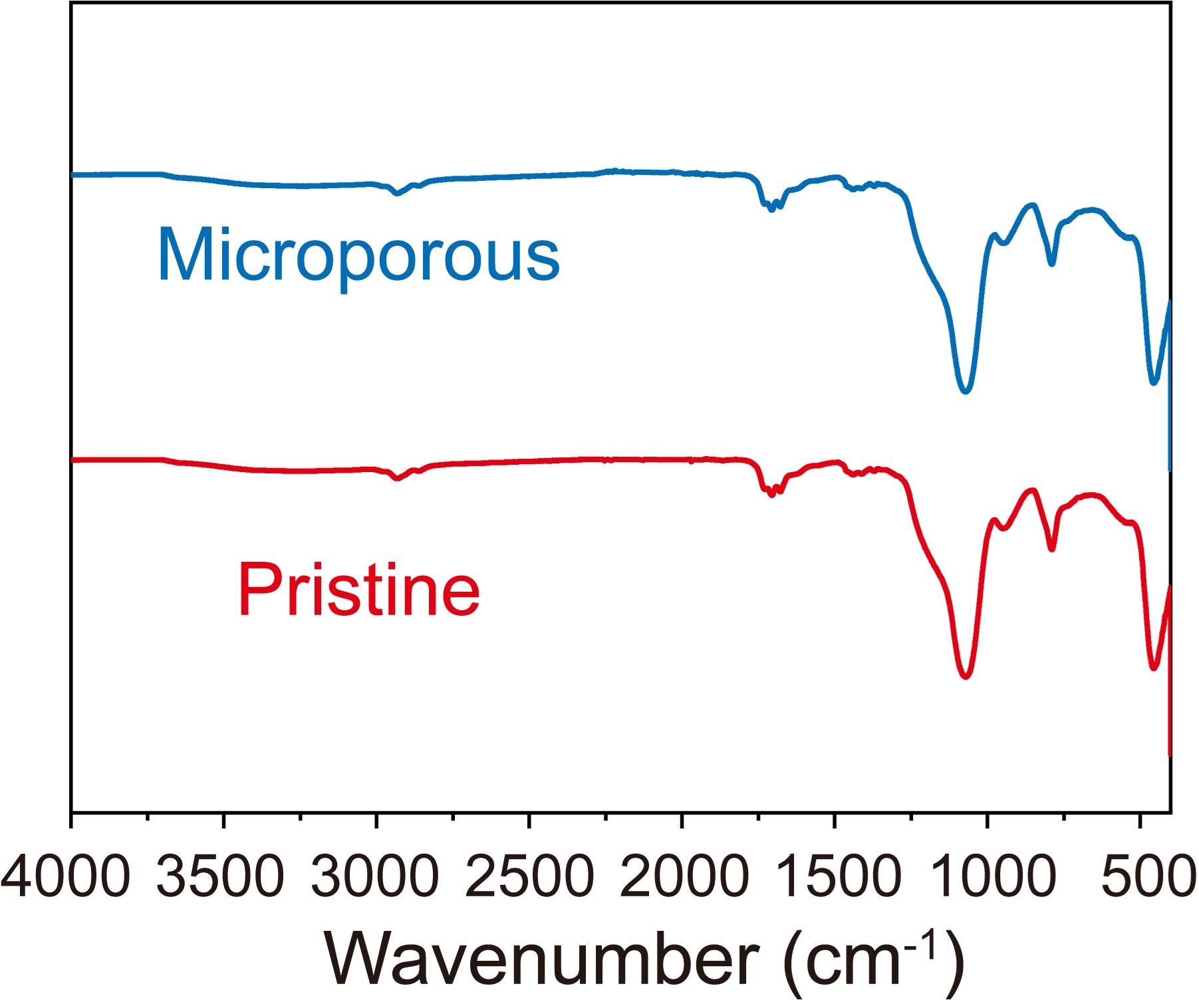


**Fig. S13** ATR-FTIR spectra of the composite before and after microporous structuring


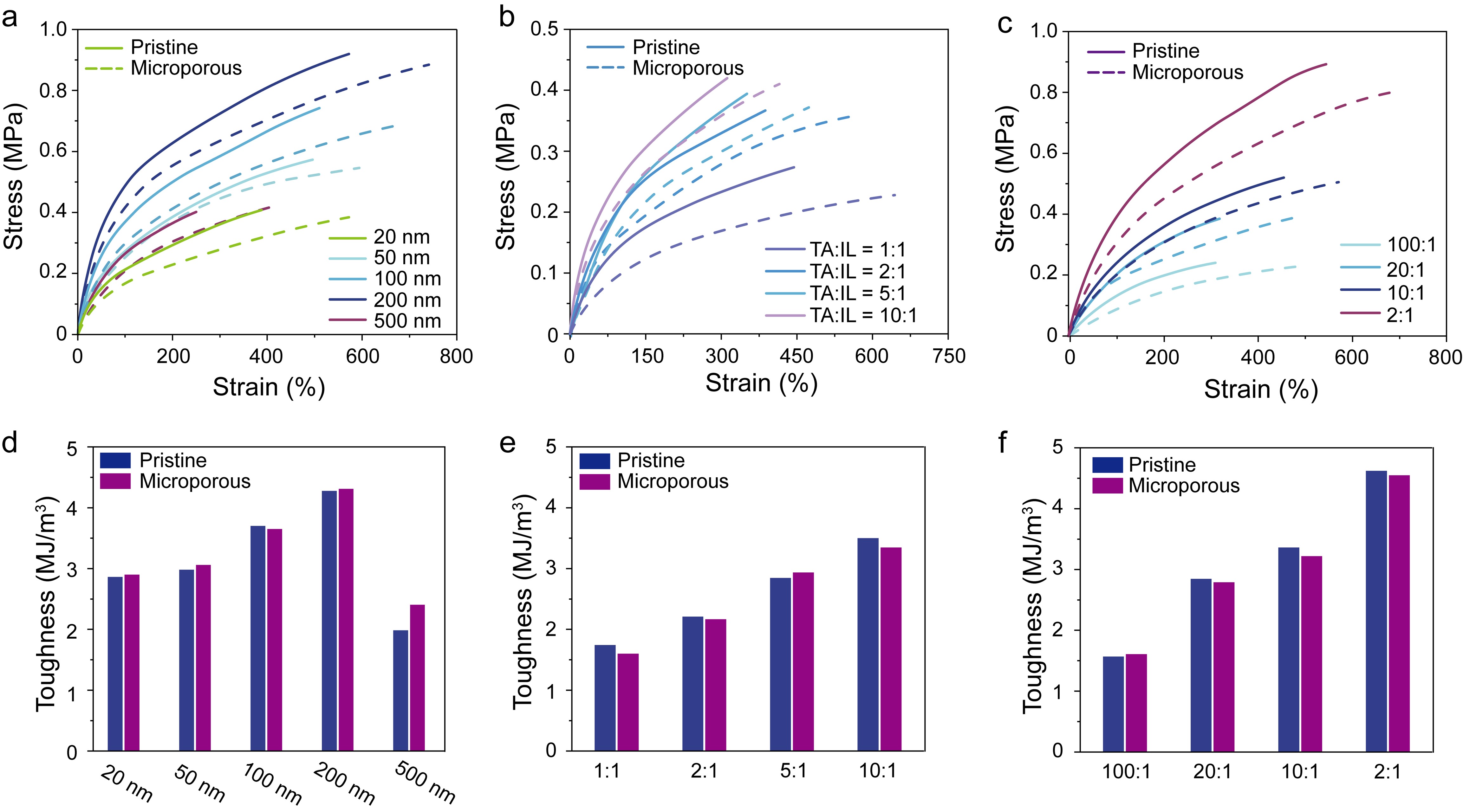


**Fig. S14** Stress-strain curves of the composites with **a** different SiO_2_-LA particle size, **b** different ionic liquid content, and **c** different SiO_2_-LA amount, and **d-f** their corresponding toughness

**
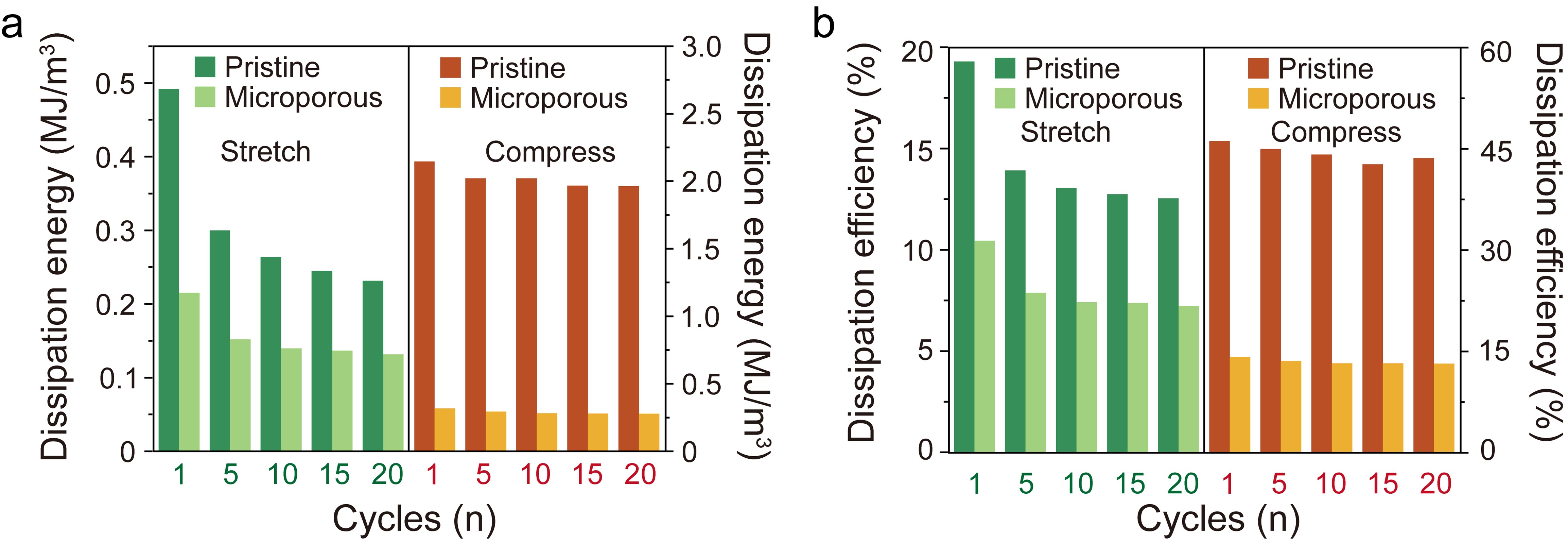
**

**Fig. S15 a** Stress dissipation and **b** dissipation efficiency during 20 cycles of 200% tensile strain and 50% compressive strain


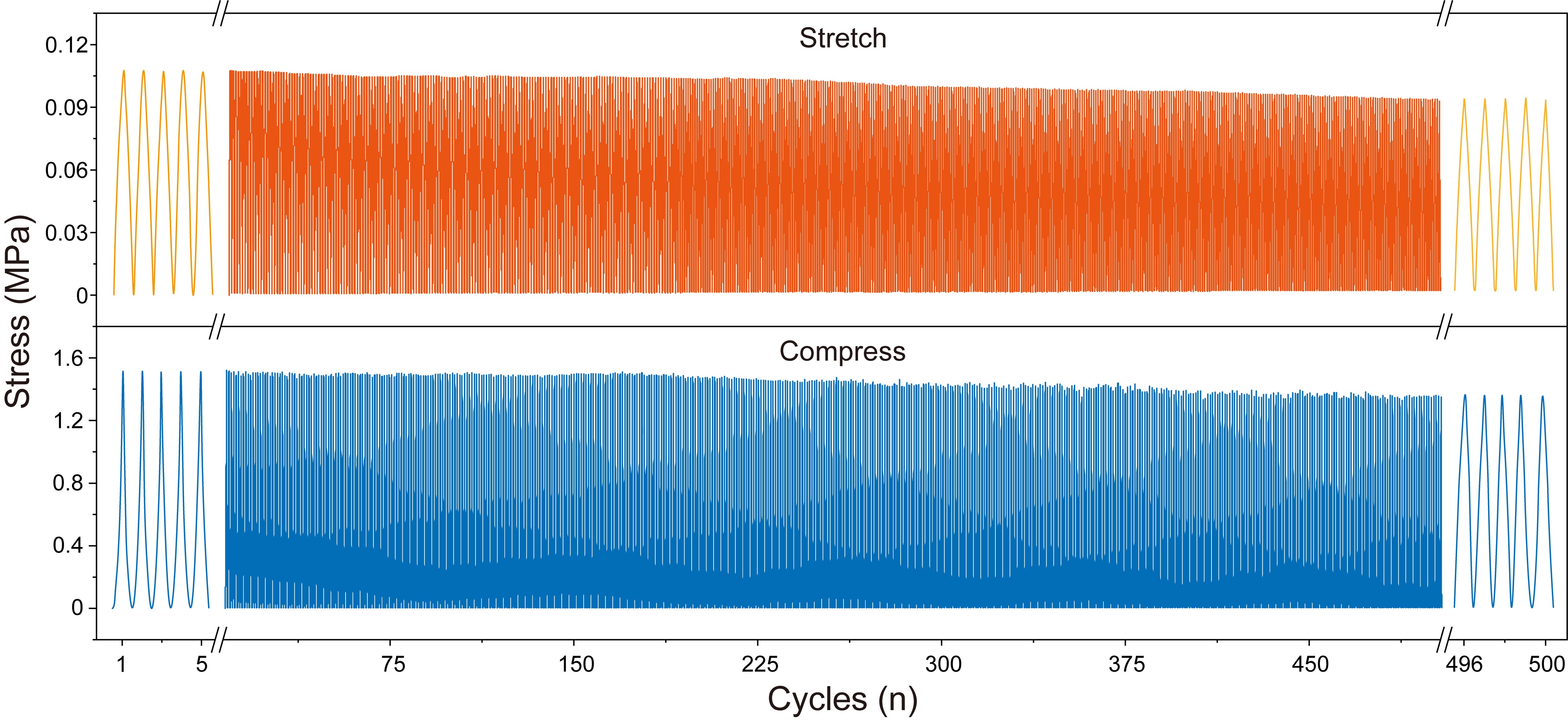


**Fig. S16** Stress variation of the composite during 500 cycles at 100% tensile strain and 50% compressive strain


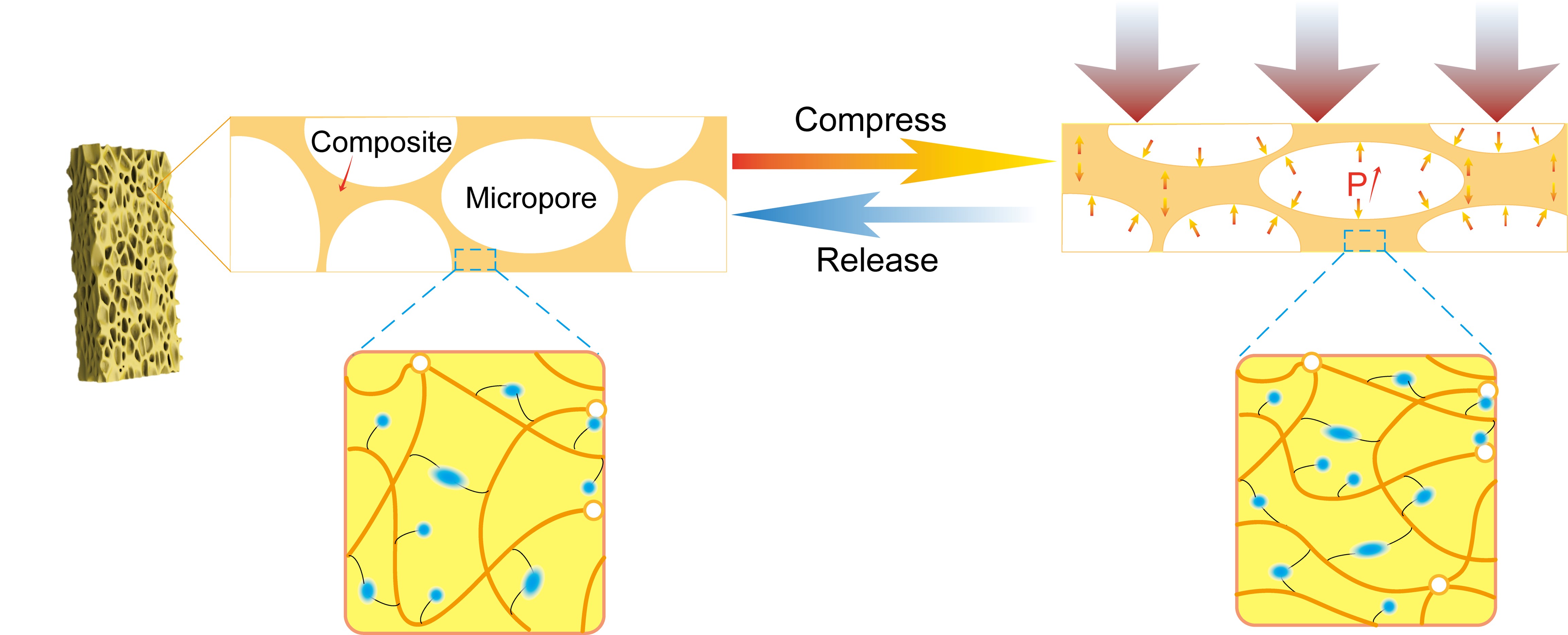


**Fig. S17** Schematic representation of the elastic recovery behavior of the microporous composite

**
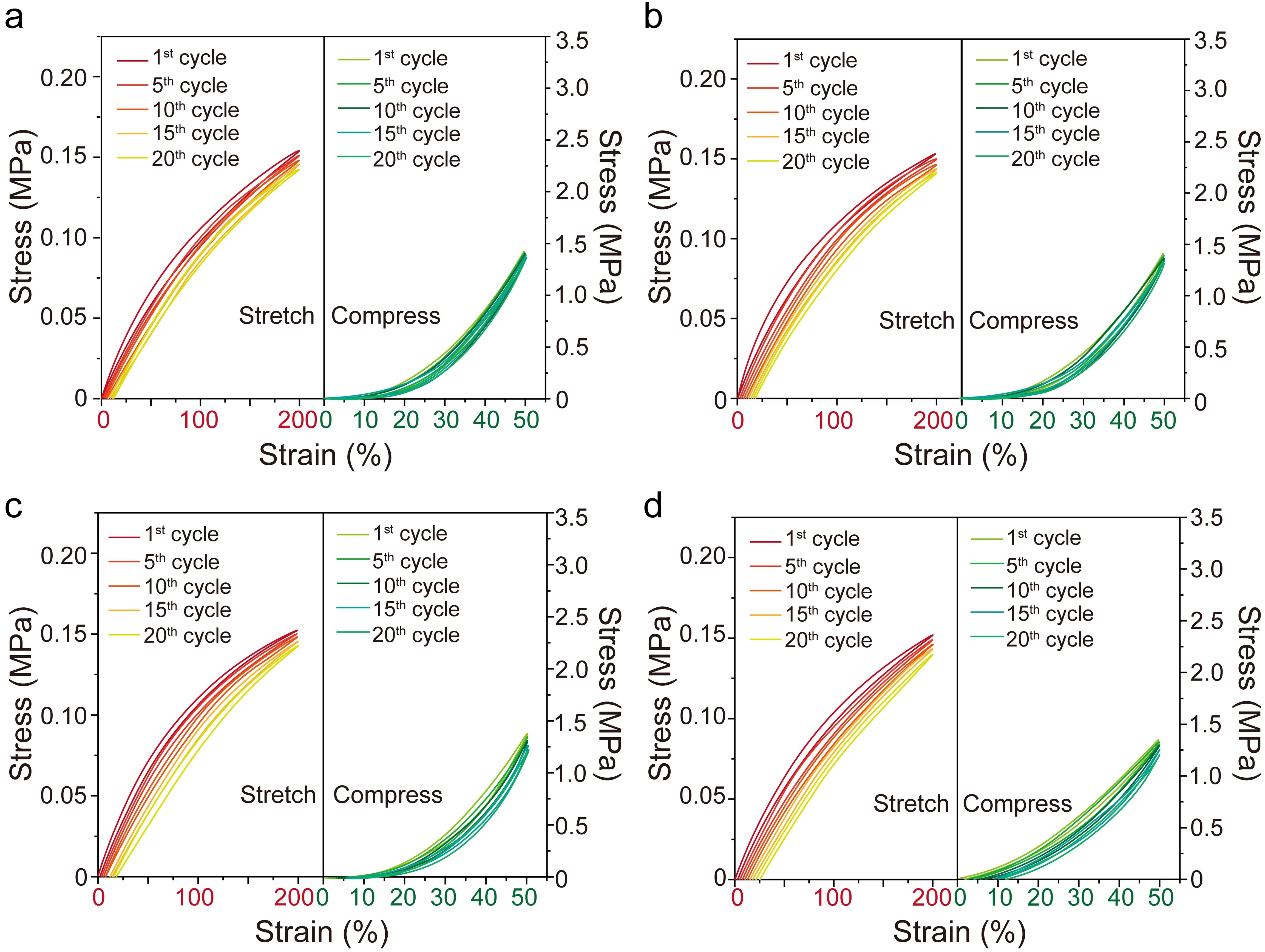
**

**Fig. S18** Stress–strain curves under cyclic loading at 200% tensile strain and 50% compressive strain for dense and microporous composites stored at ambient conditions for **a** 7, **b** 15, and **c** 30 and **d** 60 days


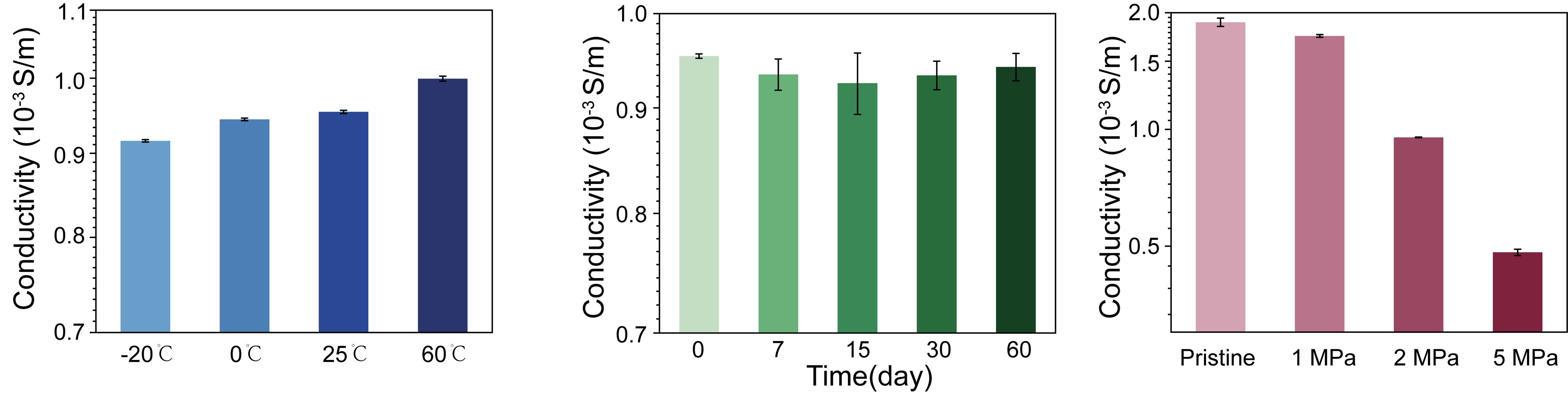


**Fig. S19** Conductivity variation of the composites as a function of foaming pressure


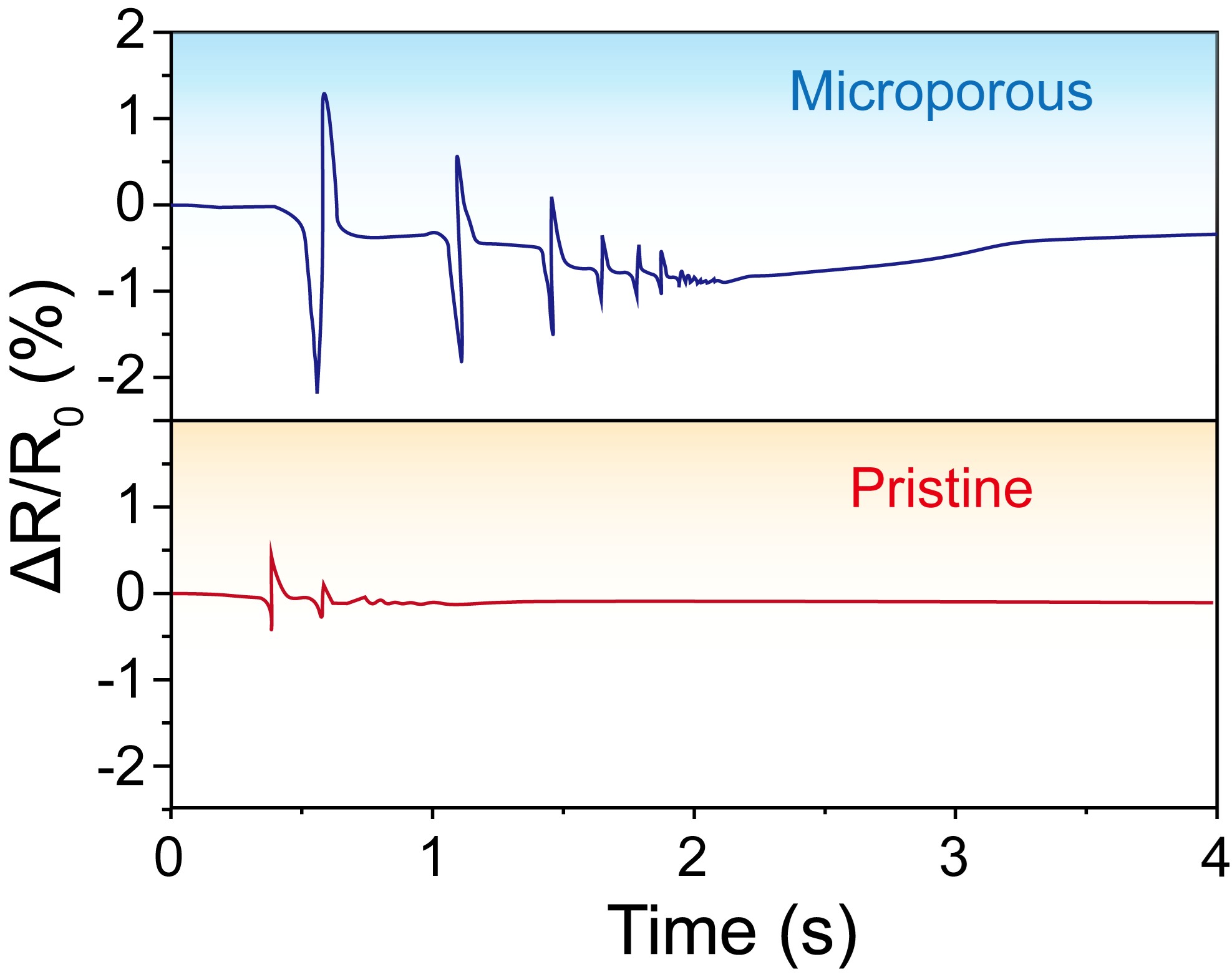


**Fig. S20** The relative resistance curves of the microporous and dense composites during the drop of a ping-pong ball


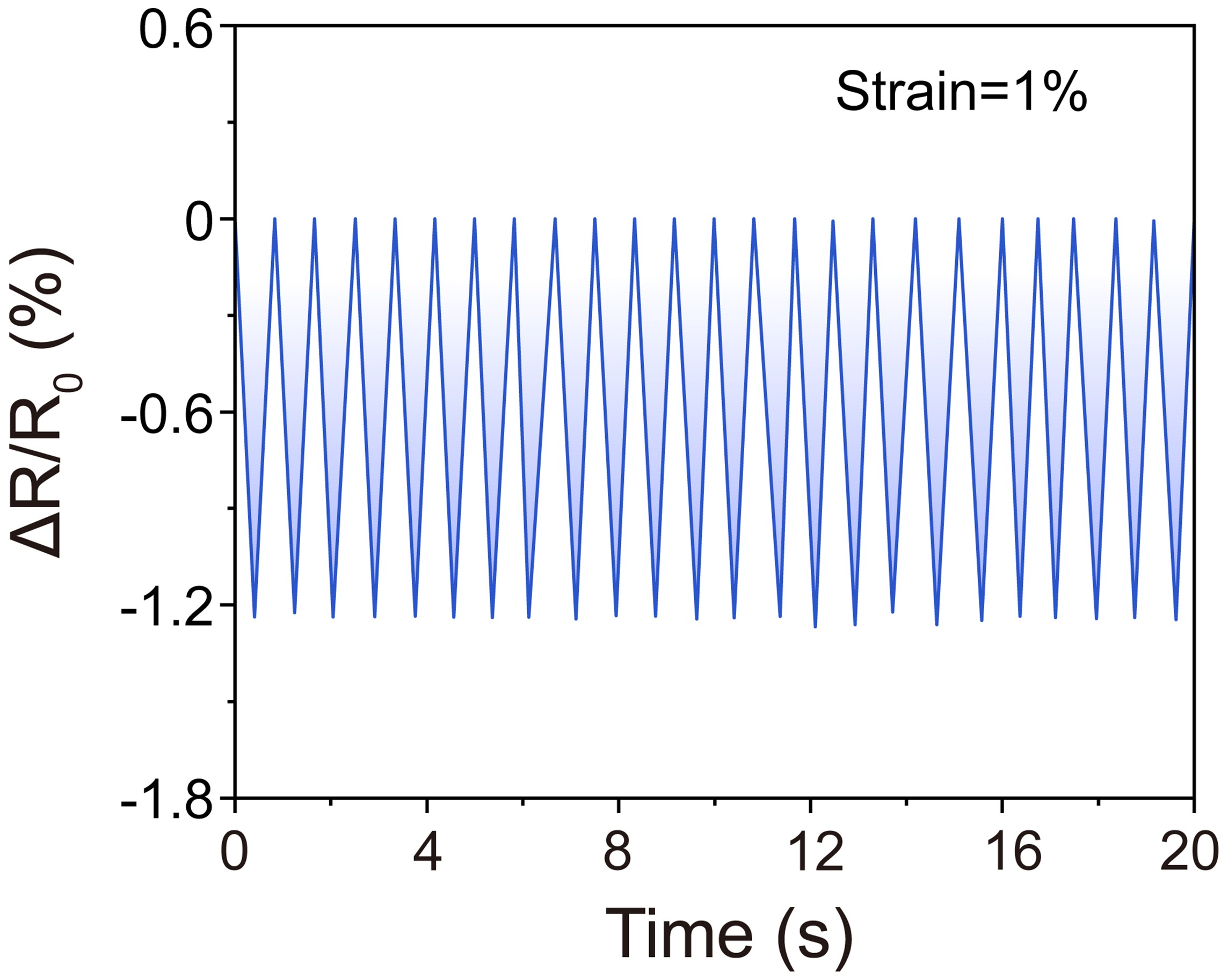


**Fig. S21** Relative resistance curves of the microporous composite under repeated 1% compressive strain


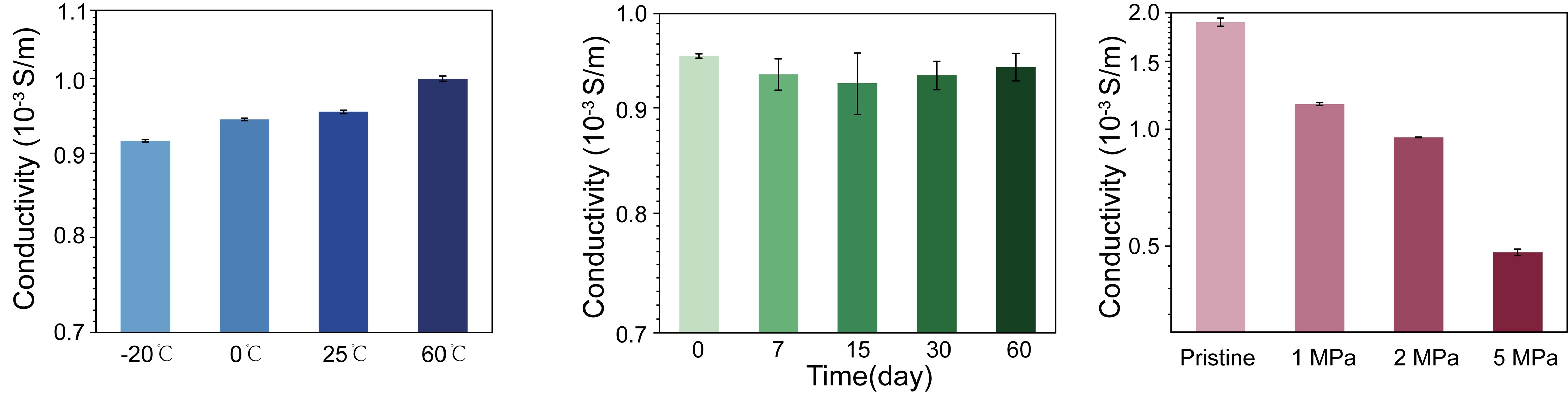


**Fig. S22** Variation in conductivity of the microporous composites with temperature


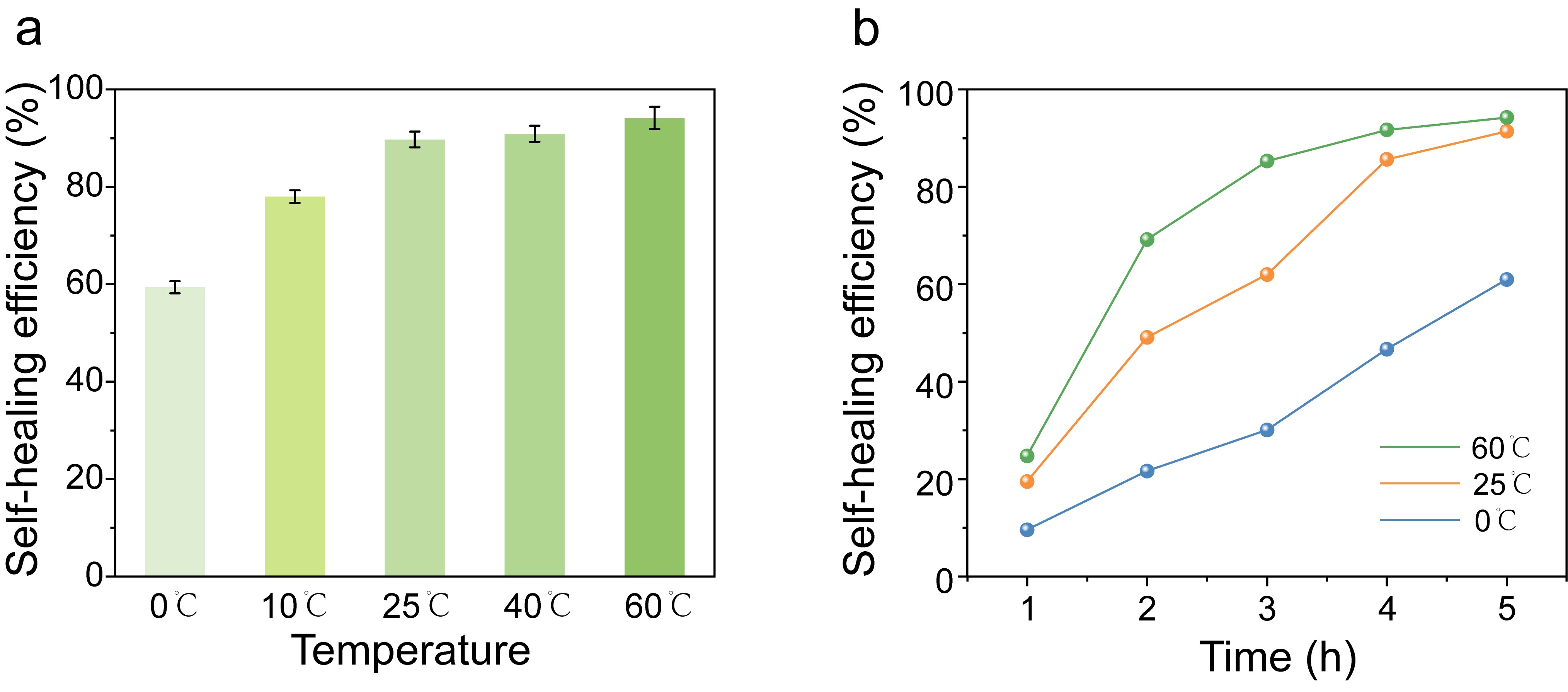


**Fig. S23** **a** Self-healing efficiency of the composites after 5 h as a function of temperature. **b** Self-healing efficiency over time at different temperatures


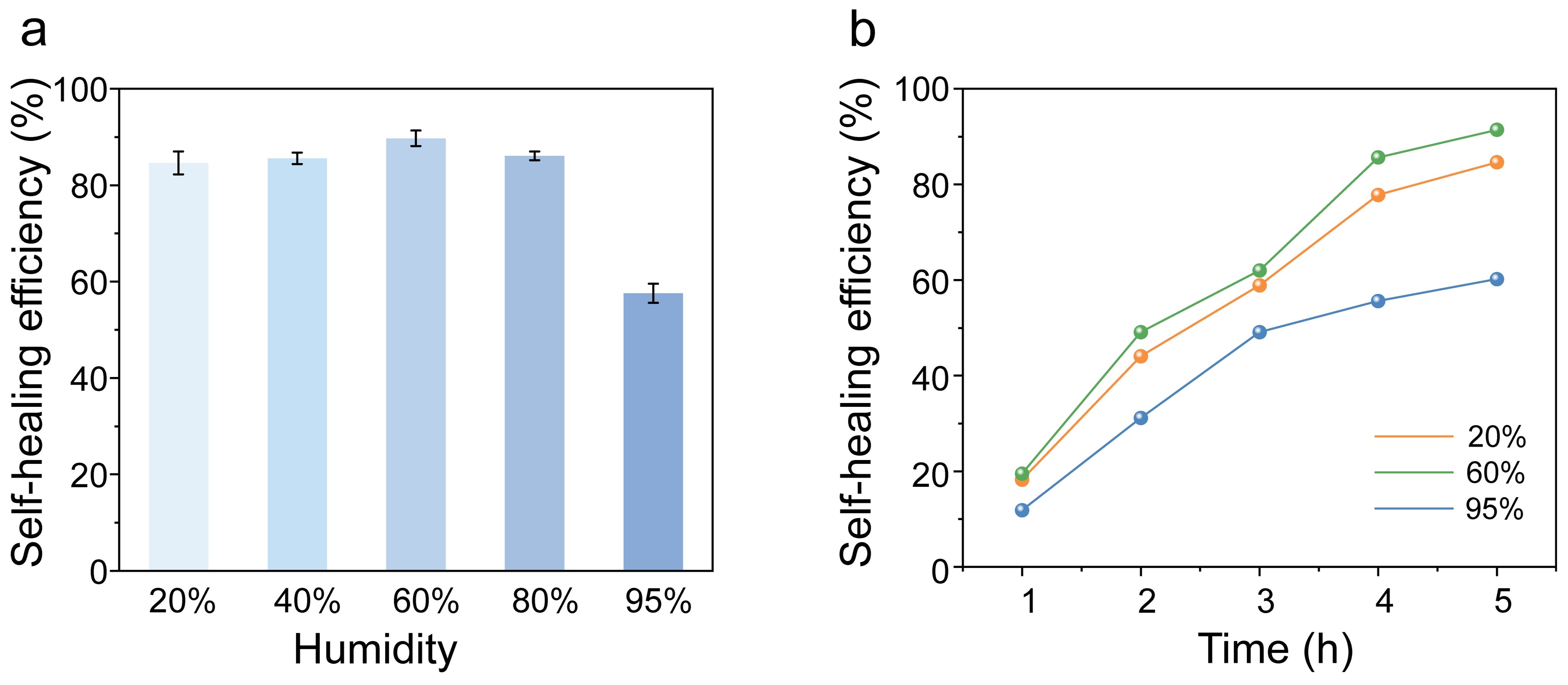


**Fig. S24** **a** Self-healing efficiency of the composites after 5 h as a function of humidity. **b** Self-healing efficiency of the composites at different humidity levels over time


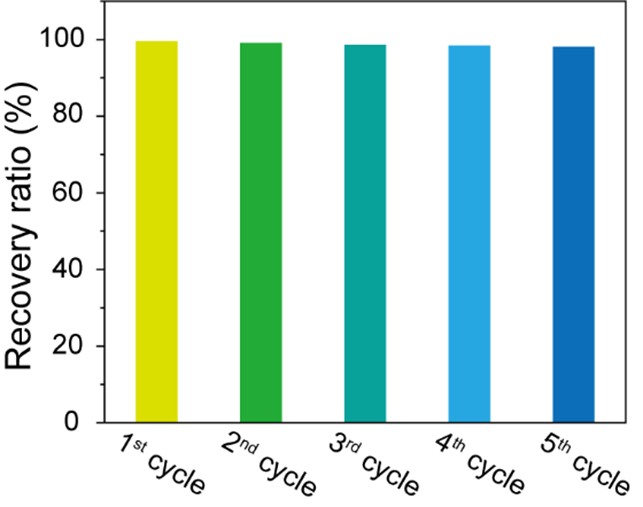


**Fig. S25** SiO_2_-LA recovery efficiency at different cycle numbers


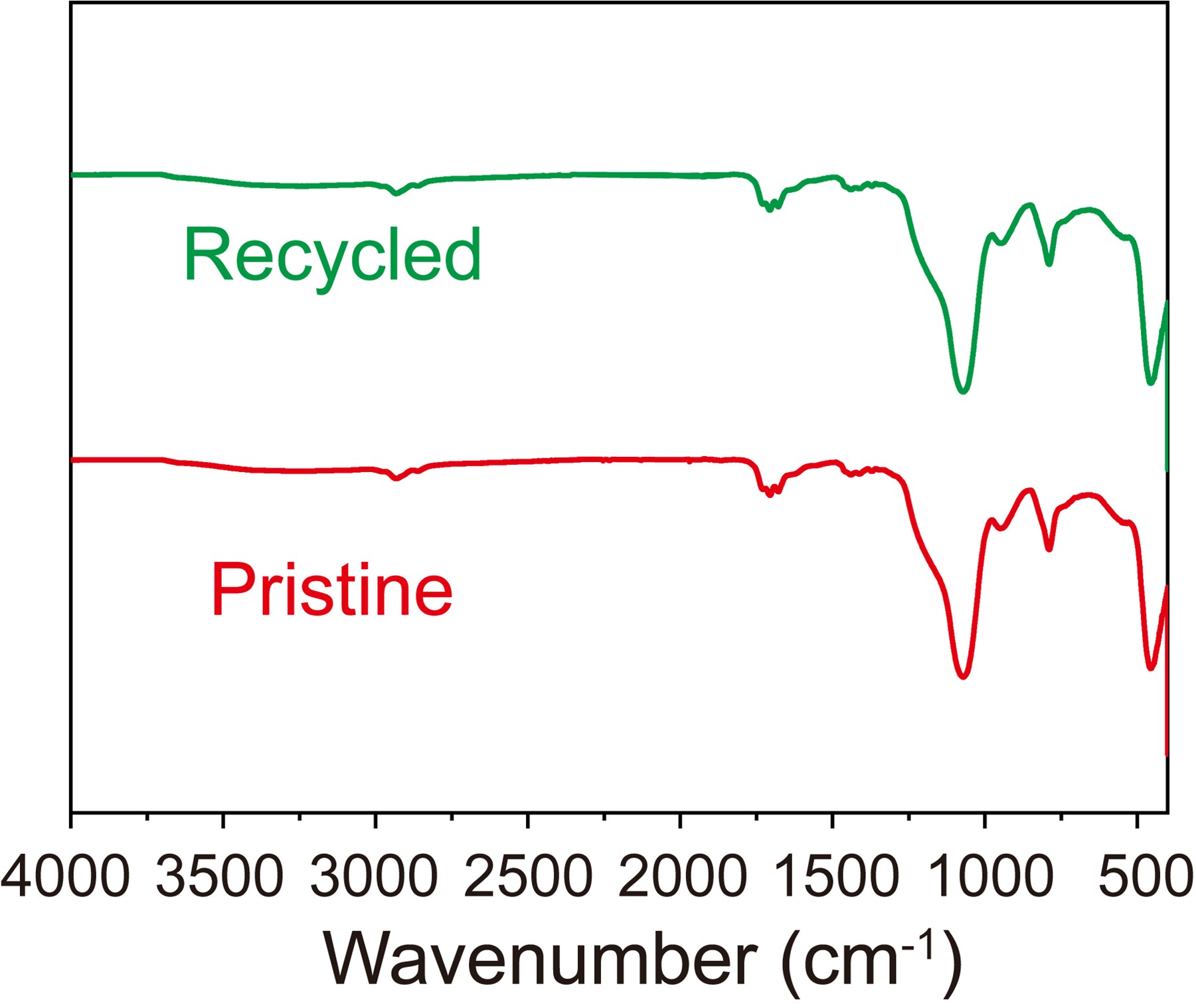


**Fig. S26** FTIR spectra of SiO_2_-LA before and after five recycling cycles

**Table. S1** Performance comparison with peer-reported elastic conductive composites.

| **Ref** | **Density**  **(g/cm^3^)** | **Self-healing**  **Efficiency (%)** | **GF** | **Recyclability** | **Recovery**  **Rate (%)** | | **Porosity(%)** |
| --- | --- | --- | --- | --- | --- | --- | --- |
| **This work** | 0.3 | 90 | 4.8 | Yes | | 99 | 81 |
| **[S1]** | 1.31 | 86 | 2.81 | No | | 75 | - |
| **[S2]** | 1.27 | 0 | 3.15 | Yes | | 84 | - |
| **[S3]** | 1.33 | 0 | 1.85 | Yes | | 85 | - |
| **[S4]** | 1.46 | 55 | 2.64 | No | | 60 | - |
| **[S5]** | 0.4 | 0 | 2.25 | No | | - | 77 |
| **[S6]** | 0.017 | 0 | 1.67 | No | | - | 96 |
| **[S7]** | 0.41 | 0 | 2.2 | No | | - | 87 |

**Supplementary References**

C. Dang, M. Wang, J. Yu, Y. Chen, S. Zhou et al., Transparent, highly stretchable, rehealable, sensing, and fully recyclable ionic conductors fabricated by one-step polymerization based on a small biological molecule. Adv. Funct. Mater. **29**(30), 1902467 (2019). https://doi.org/10.1002/adfm.201902467

C. Ma, J. Wei, Y. Zhang, X. Chen, C. Liu et al., Highly processable ionogels with mechanical robustness. Adv. Funct. Mater. **33**(31), 2211771 (2023). https://doi.org/10.1002/adfm.202211771

K.G. Cho, S. An, D.H. Cho, J.H. Kim, J. Nam et al., Block copolymer-based supramolecular ionogels for accurate on-skin motion monitoring. Adv. Funct. Mater. **31**(36), 2102386 (2021). https://doi.org/10.1002/adfm.202102386

X. Zhang, Q. Fu, Y. Wang, H. Zhao, S. Hao et al., Tough liquid-free ionic conductive elastomers with robust adhesion and self-healing properties for ionotronic devices. Adv. Funct. Mater. **34**(4), 2307400 (2024). https://doi.org/10.1002/adfm.202307400

P. Ding, J. Wang, Z. Xiao, X. Zhao, Y. Zhao et al., Low-hysteresis, high-fidelity flexible strain sensor with interconnected conductive network design for harsh environments and manipulator control. Chem. Eng. J. **511**, 162258 (2025). https://doi.org/10.1016/j.cej.2025.162258

H. Liu, X. Chen, Y. Zheng, D. Zhang, Y. Zhao et al., Lightweight, superelastic, and hydrophobic polyimide nanofiber/MXene composite aerogel for wearable piezoresistive sensor and oil/water separation applications. Adv. Funct. Mater. **31**(13), 2008006 (2021). https://doi.org/10.1002/adfm.202008006

X. Wu, Y. Han, X. Zhang, Z. Zhou, C. Lu, Large-area compliant, low-cost, and versatile pressure-sensing platform based on microcrack-designed carbon Black@Polyurethane sponge for human–machine interfacing. Adv. Funct. Mater. **26**(34), 6246–6256 (2016). https://doi.org/10.1002/adfm.201601995

| 全部文献的年份分布参考结果 by 善锋软件(R)_数据清洗与数据清除二合一小程序 | | | | | |
| --- | --- | --- | --- | --- | --- |
| 序号 | 年份 | 当年条数 | 累计条数 | 当年占比/% | 累计占比/% |
| 1 | 年份空缺 | 1 | 1 | 33 | 33 |
| 2 | 年份提取失误 | 1 | 2 | 33 | 67 |
| 3 | 年份提取失误 | 1 | 3 | 33 | 100 |

文中引文序号连续性有误：。

引文数量不同。

文中最大引文序号为： 0 < 3 (文后文献的最大/最后编号)；实际引文数量为： 0 < 3 (文后文献的最大/最后编号，即有未被引用的文献)。

文中施引和文后文献列表概况：

文中施引最末位置 = / 1 页

文中施引的位置数 = 0

文中最大引文序号 = 0

文中实际引用条数 = 0

文后文献最大编号 = 3

文后文献实际条数 = 3

文中未引文献序号 = 1，2，3

文后文献序号连续性无误。

恭喜，未发现重复的文献！
